# Horseradish Peroxidase Immobilized onto Mesoporous Magnetic Hybrid Nanoflowers for Enzymatic Decolorization of Textile Dyes: A Highly Robust Bioreactor and Boosted Enzyme Stability

**DOI:** 10.1021/acsomega.4c00703

**Published:** 2024-05-29

**Authors:** Büşra Bakar, Mustafa Akbulut, Fatma Ulusal, Ahmet Ulu, Nalan Özdemir, Burhan Ateş

**Affiliations:** † Biochemistry and Biomaterials Research Laboratory, Department of Chemistry, Faculty of Arts and Science, İnönü University, 44280 Malatya, Türkiye; ‡ Department of Chemistry, Faculty of Science, Erciyes University, 38280 Kayseri, Türkiye; § Department of Chemistry and Chemical Process Technologies, Vocational School of Technical Sciences, Tarsus University, 33400, Mersin, Türkiye

## Abstract

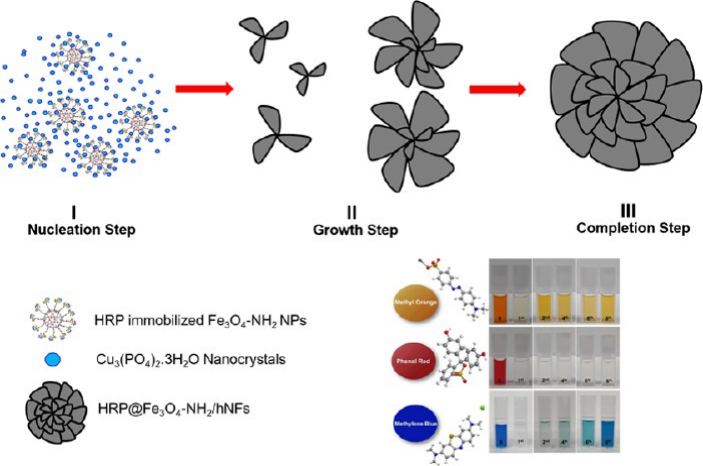

Recently, hybrid nanoflowers (hNFs), which are accepted as popular carrier supports in the development of enzyme immobilization strategies, have attracted much attention. In this study, the horseradish peroxidase (HRP) was immobilized to mesoporous magnetic Fe_3_O_4_–NH_2_ by forming Schiff base compounds and the HRP@Fe_3_O_4_–NH_2_/hNFs were then synthesized. Under optimal conditions, 95.0% of the available HRP was immobilized on the Fe_3_O_4_–NH_2_/hNFs. Structural morphology and characterization of synthesized HRP@Fe_3_O_4_–NH_2_/hNFs were investigated. The results demonstrated that the average size of HRP@Fe_3_O_4_–NH_2_/hNFs was determined to be around 220 nm. The ζ-potential and magnetic saturation values of HRP@Fe_3_O_4_–NH_2_/hNFs were −33.58 mV and ∼30 emu/g, respectively. Additionally, the optimum pH, optimum temperature, thermal stability, kinetic parameters, reusability, and storage stability were examined. It was observed that the optimum pH value shifted from 5.0 to pH 8.0 after immobilization, while the optimum temperature shifted from 30 to 80 °C. *K*_m_ values were calculated to be 15.5502 and 7.6707 mM for free HRP and the HRP@Fe_3_O_4_–NH_2_/hNFs, respectively, and *V*_max_ values were calculated to be 0.0701 and 0.0038 mM min^–1^. The low *K*_m_ value observed after immobilization indicated that the affinity of HRP for its substrate increased. The HRP@Fe_3_O_4_–NH_2_/hNFs showed higher thermal stability than free HRP, and its residual activity after six usage cycles was approximately 45%. While free HRP lost all of its activity within 120 min at 65 °C, the HRP@Fe_3_O_4_–NH_2_/hNFs retained almost all of its activity during the 6 h incubation period at 80 °C. Most importantly, the HRP@Fe_3_O_4_–NH_2_/hNFs demonstrated good potential efficiency for the biodegradation of methyl orange, phenol red, and methylene blue dyes. The HRP@Fe_3_O_4_–NH_2_/hNFs were used for a total of 8 cycles to degrade methyl orange, phenol red, and methylene blue, and degradation of around 81, 96, and 56% was obtained in 8 h, respectively. Overall, we believe that the HRP@Fe_3_O_4_–NH_2_/hNFs reported in this work can be potentially used in various industrial and environmental applications, particularly for the biodegradation of recalcitrant compounds, such as textile dyes.

## Introduction

In recent years, rapid economic growth and development have brought about increased water pollution. The widespread use of various dyes (acidic, basic, dispersive, synthetic, and mordant dyes) and phenolic compounds in the textile industry is the main cause of water pollution and is very harmful to aquatic life and human health.^1^ Synthetic dyes have low biodegradability and high chemical stability in water due to their complex aromatic structure. These properties cause the dyes to resist degradation by physicochemical methods.^2,3^ It is also worth noting that degradation products of dye should not be considered less toxic than the initial dye. Biological methods, among the existing technologies in the removal of dyes from textile wastewater, are attracting increasing attention due to their environmental friendliness.^4^ Enzymatic methods have increased their popularity with their high efficiency, low cost, substrate specificity, and lack of secondary pollution compared to other methods.^5,6^ One of the enzymes commonly used in dye removal from wastewater is peroxidases.^2^ The denature of soluble enzymes as a result of their sensitivity to industrial conditions prevents the complete degradation of the dyes. Immobilization technology is an environmentally friendly and highly effective approach to dye removal from wastewater.^7^ Immobilization is a technique designed for the purpose of facilitating enzyme recovery and, if the enzyme retains its activity, enabling the reuse of these valuable biocatalysts that were initially costly to employ.^8^ Moreover, the immobilization technique not only enhances the stability of the enzyme but also, when the immobilization protocol is meticulously crafted, serves as a tool that significantly enhances various properties of the enzyme, including selectivity and specificity.^9−11^ Also, when appropriately designed, enzyme immobilization can be integrated with the purification process of the enzyme.^8,12^ However, developing an effective immobilization protocol requires addressing multiple challenges associated with enzyme limitations. These include ensuring stability and activity under divergent conditions enhancing enzyme selectivity and specificity with distinct substrates maintaining enzyme purity, sensitivity to inhibition, and bolstering resistance to chemicals.^10^

Peroxidases (EC 1.11.1.X.) are heme-containing enzymes produced from organisms such as plants, animals, and bacteria, and they are in the oxidoreductase enzyme class.^13^ It has been reported that 87% of heme-containing peroxidases are not of animal origin, and these peroxidases are divided into three classes: class I, II, and III. Peroxidases in class I are effective in clearing and eliminating intracellular hydrogen peroxide. Class II peroxidases are involved in the degradation of lignin obtained from plants, and class III peroxidases are in the plant-derived peroxidases class.^14^ They have the ability to catalyze the oxidation of various chemical compounds using hydrogen peroxide as a cosubstrate.^14^ First, the peroxidase enzyme reacts with H_2_O_2_, and H_2_O_2_ is reduced to water, while the enzyme is oxidized to the organic cation radical (Compound 1) located on an oxyferryl center. Then, the cation radical is reduced to form a compound that protects the oxyferryl center (Compound 2), and the substrate molecule undergoes oxidation. Compound 2 oxidizes another organic substrate, releasing a new organic radical. In this way, organic radicals formed during the formation of enzymatic activity are involved in the breakdown of most pollutants.^15^

Horseradish peroxidase (HRP, EC 1.11.1.7) is one of the most popular enzymes among the class III peroxidases. Although they have a structure similar to that of Class II peroxidases, they play a role in the hydroxylation of various chemicals, unlike the peroxidative cycle. The application areas of peroxidases in this class are specific in terms of their substrates and can successfully degrade various phenolic compounds, including pyrogallol, guaiacol, catechin, and catechol.^16^ It is a commercial enzyme used in many industrial fields due to its low molecular weight (44 kDa), broad substrate specificity, and high catalytic efficiency.^17^ HRP is involved in many application areas such as the bioremediation of phenolic compounds, removal of synthetic dyes from industrial wastes, cancer treatment, and biosensors.^18^ However, the most important factor limiting the use of HRP is its poor stability against environmental conditions, as with other enzymes in their soluble form. In addition, free enzymes have disadvantages such as sensitivity to pH and temperature, not being reusable, and not being easily separated from the reaction medium. Enzyme immobilization is the most effective and practical application to solve these problems.^9,10,19,20^ The change in the conformation of the enzyme as a result of immobilization provides the enzyme with properties such as thermal stability, activity in a wide pH range, product recovery, and reusability.^21^ As a general rule, in order to achieve high performance after immobilization, it is very important to choose the appropriate carrier support. The physical properties of the solid support affect all biochemical parameters of the immobilized enzyme.^22^

Recently, much research has focused on the development of nanostructured carrier supports in enzyme immobilization.^23,24^ The HRP enzyme was immobilized on different carrier supports such as calcium/alginate beads, amino-functionalized zirconium(IV)-based metal–organic framework, chitosan and chitosan/poly(ethylene glycol) nanoparticles (NPs), hybrid nanoflowers (hNFs), functionalized graphene oxide, magnetic NPs, and so on.^21,25−31^ In this context, hNFs, which are flower-like nanostructures formed by organic–inorganic components, are gaining increasing popularity in enzyme immobilization.^32,33^ Metal phosphates such as cobalt, copper, manganese, zinc, iron, and calcium are the components that make up the inorganic part, and proteins, plant extracts, natural polymers, and nucleic acids make up the organic part.^34^ hNFs have a higher surface area than most conventional carrier supports and exhibit layered topographic features that are advantageous to the flower structure. The mild synthesis conditions of hNFs make the immobilization process quite suitable for the synthesis of enzymes. Unlike other traditional immobilization methods, in this method, most immobilized enzymes exhibit high activity and improved stability by taking advantage of the interaction between the metal ion and the enzyme molecule.^35,36^ The increase in activity varies depending on the type of metal ion and enzyme immobilized.^37^ However, hNFs are generally not easily separated from the reaction medium and are not reusable, limiting their use in most applications. To overcome this limitation, magnetic NPs that offer easy separation, high magnetization properties, and low cost are incorporated into hNFs structures.^38^ The inclusion of magnetic NPs in hNFs increases the stability of enzymes in the immobilized form and provides easy and rapid separation from the reaction medium. In addition, magnetic hNFs provide high mechanical strength and more binding area to the enzyme, greatly increasing the activity. By incorporation of magnetic NPs into hNFs, it acts as an effective carrier in the growth of hNFs leaves and contributes to the robust enzymatic stability.^39^

In this study, HRP@Fe_3_O_4_–NH_2_/hNFs were synthesized and characterized. The procedures for this purpose were carried out in three steps. For this purpose, first, magnetic mesoporous Fe_3_O_4_ NPs were prepared. After that, the synthesized magnetic mesoporous Fe_3_O_4_ NPs were functionalized with amino functional group (Fe_3_O_4_–NH_2_) using 3-aminopropyl triethoxysilane (APTES). Second, the HRP enzyme was immobilized to amino-functionalized mesoporous magnetic Fe_3_O_4_ NPs (HRP@Fe_3_O_4_–NH_2_) by the adsorption method. Third, HRP@Fe_3_O_4_–NH_2_/hNFs were synthesized using HRP@Fe_3_O_4_–NH_2_ and Cu(II) ions. Structural and morphology characterizations of the synthesized HRP@Fe_3_O_4_–NH_2_/hNFs were investigated by Fourier transform infrared (FTIR) spectroscopy, X-ray diffraction (XRD), scanning electron microscopy (SEM), vibrating-sample magnetometry (VSM), energy-dispersive X-ray spectroscopy (EDS), and dynamic light scattering (DLS). Moreover, biochemical parameters such as optimum pH, optimum temperature, kinetic parameters (*K*_m_, *V*_max_), thermal stability, reusability, and storage stability of HRP@Fe_3_O_4_–NH_2_/hNFs were determined and compared concerning its equivalent free counterpart. Finally, the effectiveness of the developed HRP@Fe_3_O_4_–NH_2_/hNFs on an industrial scale was employed in three model dyes, methyl orange (MO, an azo dye, 327.33 g mol^–1^), phenol red (PR, trimethylmethane dye, 354.38 g mol^–1^), and methylene blue (MB, a thiazine dye, 319.85 g mol^–1^), which have comparable molecular weights but different chemical structures. To the best of our knowledge, our study is the first report in which commercial HRP was immobilized as hNFs using magnetic mesoporous Fe_3_O_4_ NPs (HRP@Fe_3_O_4_–NH_2_/hNFs) which is the innovative aspect of the study. Therefore, this information can provide valuable information and a foundation for future research efforts. Additionally, its use in the enzymatic removal of textile dyes, such as MO, PR, and MB, represents an important contribution to this field. Taken together, the findings of this study aim to develop new and effective biotechnological approaches for the removal of environmental pollutants, such as textile dyes.

## Results and Discussion

### Material Characterization

The Brunauer–Emmett–Teller (BET) isotherm of mesoporous magnetic Fe_3_O_4_–NH_2_ NPs is shown in Figure S1. In addition, BET surface area, Barrett–Joyner–Halenda (BJH) average pore diameter, and pore volume of mesoporous magnetic Fe_3_O_4_ and Fe_3_O_4_–NH_2_ NPs are listed in Table 1. BET pore diameters of mesoporous magnetic Fe_3_O_4_ and Fe_3_O_4_–NH_2_ NPs were found to be 2.80 and 8.71 nm, respectively. Based on both the shape of the isotherm and the pore diameter, it can be said that the Type IV isotherm was obtained and the mesoporous structure of Fe_3_O_4_–NH_2_ NPs was preserved. Additionally, it was determined that the BET surface areas of magnetic Fe_3_O_4_ and Fe_3_O_4_–NH_2_ NPs were 41.57 and 20.55 m^2^/g and that the pore volumes were 0.144 and 0.045 cm^3^/g, respectively. Moreover, the high-resolution transmission electron microscopy (HR-TEM) image of the material prepared after coating the mesoporous Fe_3_O_4_ surface with −NH_2_ using APTES (Figure S2) shows that the porous structure of the material was preserved.

**Table 1:** BET Surface Area, BJH Average Pore Diameter, and Pore Volume of Mesoporous Magnetic Fe_3_O_4_ and Fe_3_O_4_–NH_2_ NPs

sample	BET surface area (m^2^/g)	BJH average pore diameter (nm)	pore volume (cm^3^/g)
Fe_3_O_4_ NPs	41.57	2.80	0.144
Fe_3_O_4_–NH_2_ NPs	20.55	8.71	0.045

**Figure 1:**
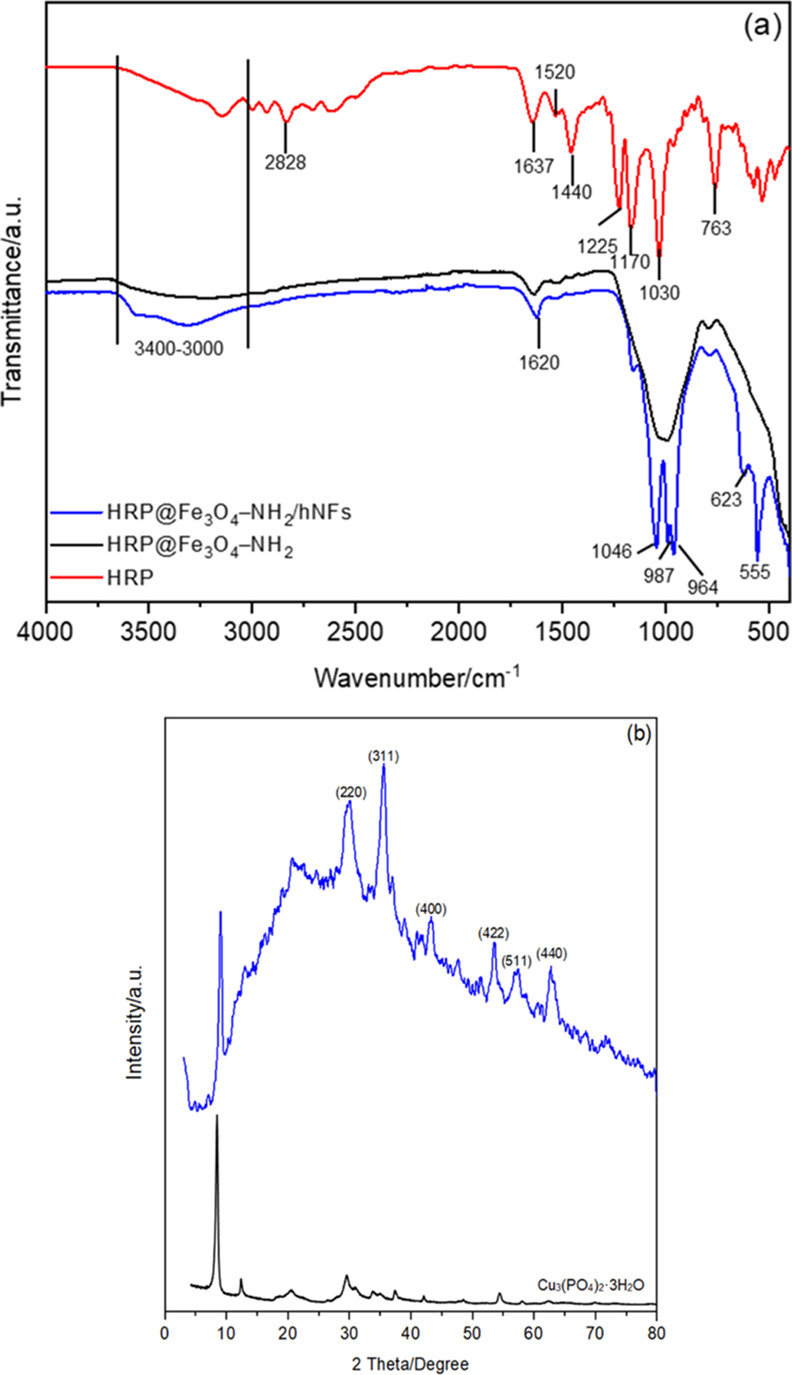
(a) FTIR spectra of HRP, HRP@Fe_3_O_4_–NH_2_, and HRP@Fe_3_O_4_–NH_2_/hNFs and (b) XRD patterns of Cu_3_(PO_4_)_2_·3H_2_O and HRP@Fe_3_O_4_–NH_2_/hNFs.

The chemical bonds of the HRP, HRP@Fe_3_O_4_–NH_2_, and HRP@Fe_3_O_4_–NH_2_/hNFs were characterized by FTIR spectroscopy. As shown in Figure 1a, peaks at 1637, 1520, and 1440 cm^–1^ were assigned to the amide I band, amide II band, and several modes of NH (amide III), respectively.^40^ Observed spectral regions at 3140 cm^–1^ were attributed to O–H vibration modes, at 2828 cm^–1^ for the alkyl (−CH_2_) chains.^41,42^ The single hydrogen of the terminal acetylene was characteristic at 770–620 cm^–1^, while aliphatic amines (C–N stretching vibration) assigned at the region 1080 cm^–1^.^43^ Besides, around 600 cm^–1^, C–H bond deformation or bending for alkynes may be indicated.^43^ For the HRP@Fe_3_O_4_–NH_2_/hNFs, the Fe–O peak was observed at 555 cm^–1^.^44−46^ The strong bands at 1046 and 987 cm^–1^ were assigned to the Si–O–Si and Si–O stretching vibrations of APTES to the surface of magnetic NPs.^47,48^ The absorption peaks at 964 cm^–1^ (symmetric stretching) and 623 cm^–1^ (bending) were ascribed to P–O vibrations and demonstrated the presence of phosphate groups.^32,35^ In addition, the band around 1620 cm^–1^ was assigned to the presence of HRP. These results confirmed that Fe_3_O_4_–NH_2_/hNFs were successfully synthesized and HRP was immobilized on Fe_3_O_4_–NH_2_/hNFs.

XRD patterns of the HRP@Fe_3_O_4_–NH_2_/hNFs and Cu_3_(PO_4_)_2_·3H_2_O are illustrated in Figure 1b. It was revealed that the XRD pattern of the HRP@Fe_3_O_4_–NH_2_/hNFs showed 2θ angles of 30.2, 35.6, 43.2, 53.6, 57.2, and 62.7°, which correspond to (220), (311), (400), (422), (511), and (440) orientations, respectively. The pattern matched well with those of Cu_3_(PO_4_)_2_·3H_2_O (JCPDS card; 00-022-0548) and Fe_3_O_4_ (JCPDS card; 19-0629) characteristic peaks (30.1°, (220), 35.5° (311), 43.1° (400), 57.0° (511), and 62.9° (440)).^29^ XRD results confirmed that the presence of HRP did not change the position of the peaks, which indicates that immobilization of HRP does not change the crystal structure of the Fe_3_O_4_–NH_2_/hNFs.

**Figure 2:**
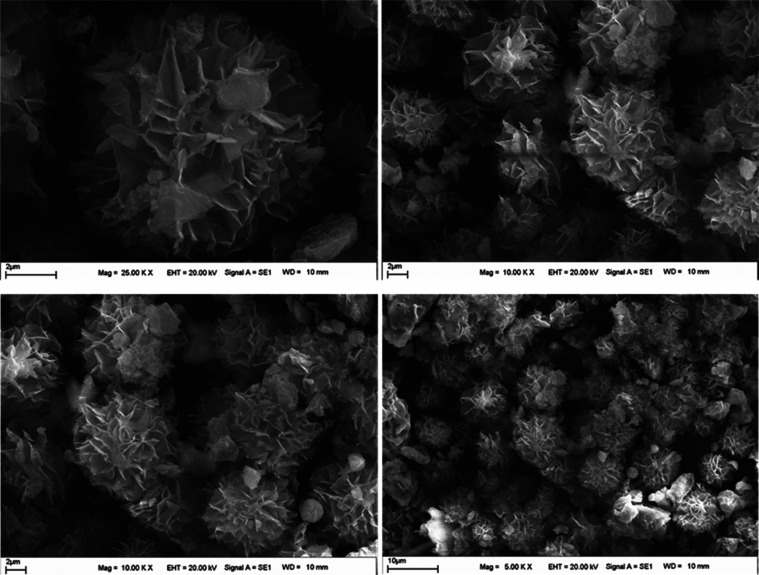
SEM images of the HRP@Fe_3_O_4_–NH_2_/hNFs in different magnifications.

As previously mentioned in the last part of the Introduction section, before synthesizing HRP@Fe_3_O_4_–NH_2_/hNFs, synthesized magnetic mesoporous Fe_3_O_4_ NPs were functionalized with an amino functional group (−NH_2_). After functionalization, the HRP enzyme was immobilized onto the Fe_3_O_4_–NH_2_ by forming Schiff base compounds.^49^ Schiff base compound formation or cross-linking between enzyme molecules and Fe_3_O_4_–NH_2_ not only stabilizes the enzyme in active conformation but also averts the enzyme leaching from the magnetic carrier. For this reason, amino-functionalized magnetic Fe_3_O_4_–NH_2_ NPs are widely used for enzyme immobilization. After all, HRP@Fe_3_O_4_–NH_2_/hNFs were synthesized by using HRP@Fe_3_O_4_–NH_2_ and Cu(II) ions. For this purpose, HRP-immobilized mesoporous Fe_3_O_4_–NH_2_ NPs were dissolved in phosphate-buffered saline (PBS) and a certain amount of Cu(II) ions was added. This mixture was incubated for 3 days. In principle, the formation of flower-like structures of hNFs can be explained in three stages: nucleation, growth, and completion. During the incubation period, first primary Cu_3_(PO_4_)_2_ nanocrystals are formed as a result of the nucleation of Cu(II) ions and (PO_4_)^3–^ ions. The resulting Cu_3_(PO_4_)_2_ nanocrystals can form complexes with the amine or amide moieties of HRP-immobilized mesoporous Fe_3_O_4_–NH_2_ NPs mainly through coordination interaction. Under certain incubation conditions, such interactions between the enzyme molecules and Cu_3_(PO_4_)_2_ nanocrystals allow the formation of highly branched flower-like hybrid structures with a high surface-to-volume ratio. The morphology of the synthesized HRP@Fe_3_O_4_–NH_2_/hNFs was observed using SEM images in different magnifications (Figure 2). SEM images show the existence of a self-assembled hierarchical flower morphology. It appears that HRP@Fe_3_O_4_–NH_2_/hNFs have uniform structures with good monodispersed. Additionally, it is seen the HRP-immobilized magnetic NPs do not change the nanoflower morphology.^50,51^

**Figure 3:**
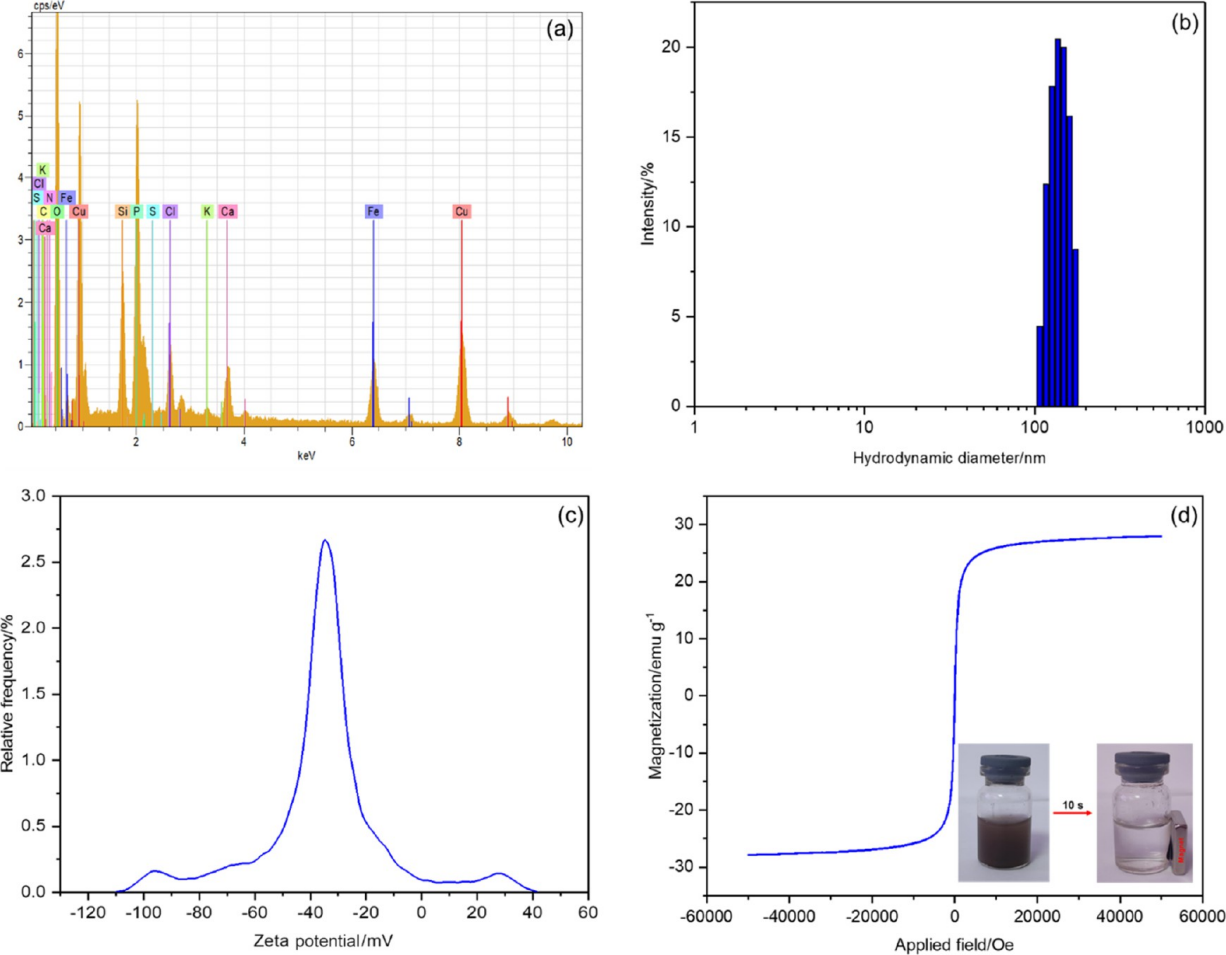
(a) EDS pattern, (b) hydrodynamic diameter distribution, (c) ζ-potential, and (d) VSM curve of the HRP@Fe_3_O_4_–NH_2_/hNFs. The inset photo shows the HRP@Fe_3_O_4_–NH_2_/hNFs in water attracted to the vial wall by a magnet.

The EDS analysis of the HRP@Fe_3_O_4_–NH_2_/hNFs is shown in Figure 3a. Besides, Figure S3 also shows EDS elemental mapping images. The copper (Cu), iron (Fe), sulfur (S), carbon (C), nitrogen (N), silicon (Si), phosphorus (P), and oxygen (O) elements were found in the HRP@Fe_3_O_4_–NH_2_/hNFs. Among these elements, Fe, N, O, Si, P, and Cu elements were derived from Fe_3_O_4_–NH_2_ and Cu_3_(PO_4_)_2_. The C, N, and S elements were from HRP confirming the successful immobilization of HRP into the Fe_3_O_4_–NH_2_/hNFs.

To better understand the particle size and surface charge of the HRP@Fe_3_O_4_–NH_2_/hNFs in the solution environment, DLS and ζ-potential measurements were performed, and the results are shown in Figure 3b,c, respectively. It is evident from Figure 3b, the DLS histogram of the HRP@Fe_3_O_4_–NH_2_/hNFs revealed that the hydrodynamic diameter (i.e., a diameter of particle with hydration shell) of the HRP@Fe_3_O_4_–NH_2_/hNFs was in the range of 200–250 nm, with an average hydrodynamic diameter of 220 ± 25 nm. It can be inferred from Figure 3c that the value of ζ-potential was found as −33.58 mV. Particles with a ζ-potential value above +30 mV or below −30 mV are generally considered colloidally stable.^52^ Accordingly, HRP@Fe_3_O_4_–NH_2_/hNFs were almost stable. The negative ζ-potential value of the HRP@Fe_3_O_4_–NH_2_/hNFs was probably due to the carboxylate anions of amino acids in the structure of immobilized HRP. Therefore, the ζ-potential value suggested that the surface of the HRP@Fe_3_O_4_–NH_2_/hNFs was negatively charged and the strong interparticle repulsion between negative charges protected the HRP@Fe_3_O_4_–NH_2_/hNFs from aggregation.^53^

The magnetic property of the HRP@Fe_3_O_4_–NH_2_/hNFs was evaluated using the magnetization curve prepared by VSM with applied field −60 000 ≤ Oe ≤ 60 000 at room temperature. According to Figure 3d, the curve was S-shaped and showed no hysteresis with negligible coercivity and remanence. The magnetization saturation value for HRP@Fe_3_O_4_–NH_2_/hNFs was ∼30 emu/g. This value was consistent with previous study results.^54,55^ Although the magnetization saturation value of the HRP@Fe_3_O_4_–NH_2_/hNFs was not very high, it still had the recognized property of being easily separated from the reaction systems. As observed in Figure 3d (inset), when an external magnetic field was applied, the HRP@Fe_3_O_4_–NH_2_/hNFs were attracted to the bottle wall within 10 s, indicating that it has ease of recycling. Besides, HRP@Fe_3_O_4_–NH_2_/hNFs were uniformly dispersed in water, forming a stable suspension.

### Biochemical Evaluation of Free HRP and HRP@Fe_3_O_4_–NH_2_/hNFs

#### Effect of pH

**Figure 4:**
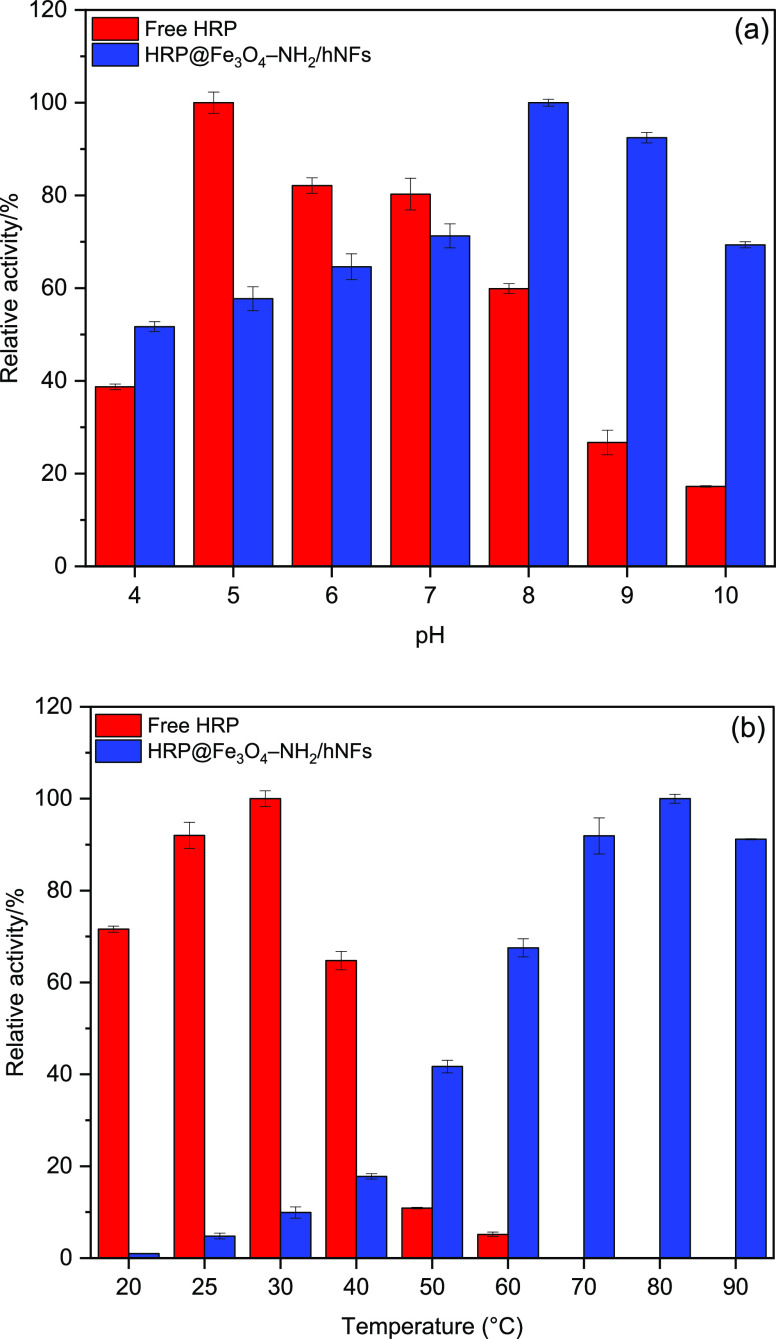
Effect of (a) pH and (b) temperature on the activities of free HRP and HRP@Fe_3_O_4_–NH_2_/hNFs.

The effect of pH on enzyme activities is very effective in many industrial applications. The relative activity of free HRP and the HRP@Fe_3_O_4_–NH_2_/hNFs at varying pHs is illustrated in Figure 4a. The free HRP and HRP@Fe_3_O_4_–NH_2_/hNFs showed maximum activity at pH 5.0 and 8.0, respectively. The optimal values may differ because immobilization alters the ionization states of protein functional groups in the microenvironment around the enzyme catalytic site, resulting in an alkaline shift in the optimal pH of the HRP@Fe_3_O_4_–NH_2_/hNFs. Nevertheless, free HRP exhibited a higher activity than the HRP@Fe_3_O_4_–NH_2_/hNFs in a certain pH range (5.0–7.0). On the other hand, it was observed that alkaline conditions increased the catalytic activity of HRP@Fe_3_O_4_–NH_2_/hNFs compared to free HRP. While free HRP showed high activity at acidic pHs, beyond the neutral pH region, the free HRP activity decreased significantly. It was observed that the enzymatic activity of HRP@Fe_3_O_4_–NH_2_/hNFs shifted sharply to basic pH values after immobilization. In the study conducted by Melo et al.,^18^ immobilized HRP enzyme maintained its activity around 70% at pH 9.0, while HRP@Fe_3_O_4_–NH_2_/hNFs maintained 70% of its activity at pH 10.0, providing a wider alkaline pH area. Mohamed et al. immobilized the HRP enzyme into magnetic NPs and determined the optimal pH as pH 7.0 for the free enzyme and pH 7.5 for the immobilized enzyme.^56^ Similarly, Karim et al. determined that the optimum pH of HRP immobilized to silver nanoparticles was 8.0.^57^ The changing enzyme activity after immobilization may be due to the properties of the carrier support and the conformational change in the catalytic region of the enzyme.^58^ Moreover, the robust composition of Fe_3_O_4_–NH_2_/hNFs could potentially offer a protective shield, akin to armor, shielding the enzyme from denaturation when subjected to challenging environmental conditions.^59^

#### Effect of Temperature and Activation Energy

The effect of the temperature on the enzymatic activities of free HRP and HRP@Fe_3_O_4_–NH_2_/hNFs is shown in Figure 4b. The activity of free HRP reached its optimum value at 30 °C. Unlike free HRP, HRP@Fe_3_O_4_–NH_2_/hNFs showed the highest activity at 80 °C. A shift in optimum temperature was observed due to hydrophobic and other secondary interactions between the enzyme and the supporting environment.^60^ Although free HRP exhibited high relative activity at lower temperatures, it did not show activity at temperatures higher than 60 °C. This steep decline happens due to enzyme denaturation caused by excessive heat. On the other hand, the HRP@Fe_3_O_4_–NH_2_/hNFs exhibited activity up to 90 °C, with a different profile from free HRP. Initially, at lower temperatures, the HRP@Fe_3_O_4_–NH_2_/hNFs exhibited lower relative activities compared to free HRP. However, the HRP@Fe_3_O_4_–NH_2_/hNFs at higher temperatures were able to retain most of their catalytic activity (90% at 90 °C). The observations made in this study are consistent with Weber et al.,^61^ in their study of the immobilization of HRP in calcium alginate-starch hybrid support, where immobilized HRP displayed 87.21 and 79.95% at 80 and 85 °C, respectively. The lower sensitivity of the HRP@Fe_3_O_4_–NH_2_/hNFs to heat can be explained by the protection provided by the Fe_3_O_4_–NH_2_/hNFs, as well as the occurrence of hydrophobic and secondary interactions between support and enzyme, which confer higher structural stability to the protein under higher temperatures and consequently preserve the activity of catalytic sites.^61^ In addition, results consistent with the literature have been obtained for free HRP, but no literature study has been found in which the optimum temperature for immobilized HRP is 80 °C. Therefore, it can be concluded that the bioreactor system proposed in this study is superior to that of other counterparts.

**Figure 5:**
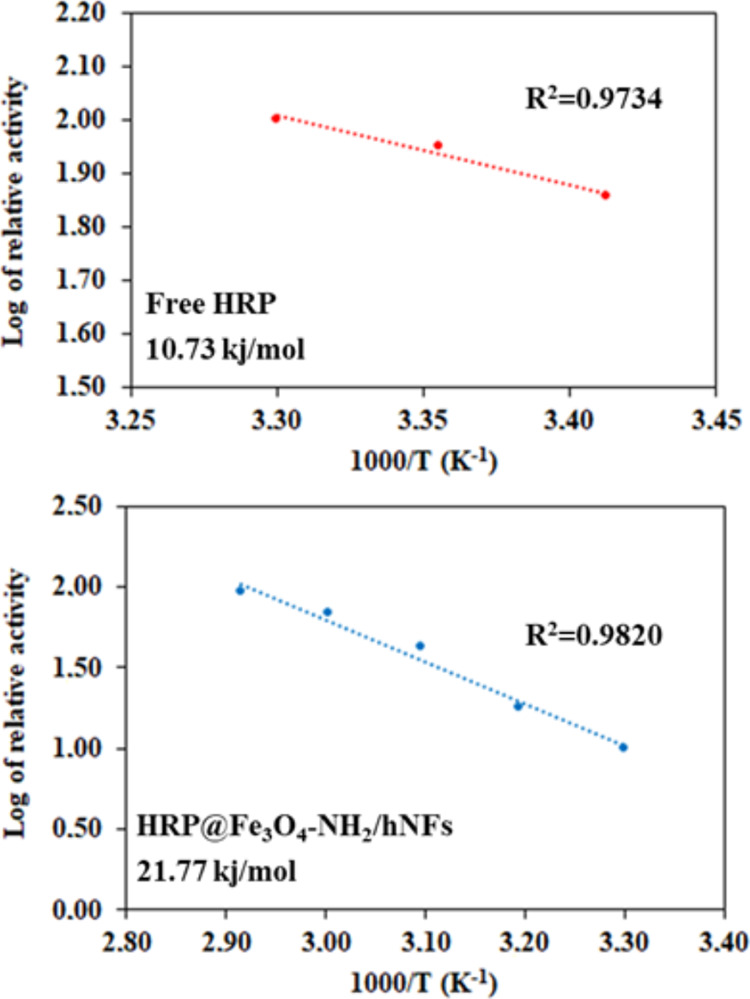
Arrhenius plots for free HRP and HRP@Fe_3_O_4_–NH_2_/hNFs.

The change in the reaction rate with temperature in the realization of enzymatic activity was determined by calculating the *E*_a_ value with the help of the Arrhenius equation. Arrhenius plots of free HRP and HRP@Fe_3_O_4_–NH_2_/hNFs are given in Figure 5. For free HRP, the *E*_a_ value was estimated as 10.73 kJ/mol and the corresponding *R*^2^ value was 0.9734, while it was estimated as 21.77 kJ/mol for HRP@Fe_3_O_4_–NH_2_/hNFs and the *R*^2^ value was 0.9820. After the immobilization process, higher catalytic efficiency is expected as a result of the lower *E*_a_ value in immobilized enzymes. However, in this study, an increased *E*_a_ value was observed in the immobilized enzyme. The reason for this is that a higher energy is required for the thermal denaturation of the immobilized enzyme.

#### Kinetic Parameters (*K*_m_ and *V*_max_)

**Figure 6:**
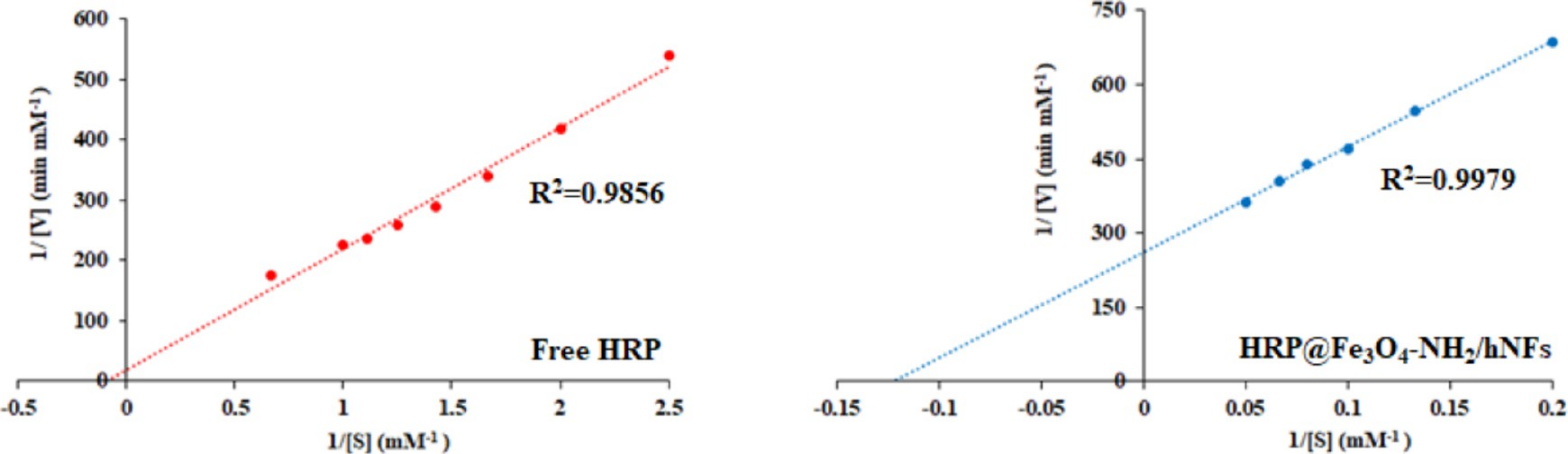
Lineweaver–Burk plots of free HRP and HRP@Fe_3_O_4_–NH_2_/hNFs.

The effect of substrate concentration on free HRP and HRP@Fe_3_O_4_–NH_2_/hNFs was achieved by changing the H_2_O_2_ concentration while keeping the guaiacol concentration constant. The Lineweaver–Burk plots are shown in Figure 6, and *K*_m_ and *V*_max_ values are listed in Table 2. While the *K*_m_ and *V*_max_ values for free HRP were found to be 15.5502 ± 0.0587 and 0.0701 ± 0.0089 mM min^–1^, these values for HRP@Fe_3_O_4_–NH_2_/hNFs were calculated as 7.6707 ± 0.0332 and 0.0038 ± 0.0001 mM min^–1^, respectively. The HRP@Fe_3_O_4_–NH_2_/hNFs have lower *K*_m_ and *V*_max_ values than the free enzyme. The lower *K*_m_ value of HRP@Fe_3_O_4_–NH_2_/hNFs compared to free HRP indicates that the immobilized enzyme has a higher affinity toward its substrate. In other words, the conformational change that occurred after immobilization enabled the enzyme to interact with the substrate more easily.^62^ The interaction and conformational change between the enzyme and the carrier support may increase, decrease, or not change the *K*_m_ and *V*_max_ values. This phenomenon occurs due to conformational changes in the enzyme, electrostatic interaction between the enzyme and the environment, the nature of the microenvironment, and the supporting environment.^63^ Various works involving the immobilization of peroxidase enzymes in different supports have reported similar changes in kinetic parameters after immobilization, such as those of Weber et al.,^61^ Kalsoom et al.,^60^ Bilal and Asgher.^64^

**Table 2:** Kinetic Parameters of Free HRP and HRP@Fe_3_O_4_–NH_2_/hNFs

catalyst	*K*_m_ (mM)	*V*_max_ (mM min^–1^)	*R*^2^
free HRP	15.5502 ± 0.0587	0.0701 ± 0.0089	0.9856
HRP@Fe_3_O_4_–NH_2_/hNFs	7.6707 ± 0.0332	0.0038 ± 0.0001	0.9979

#### Thermal Stability

**Figure 7:**
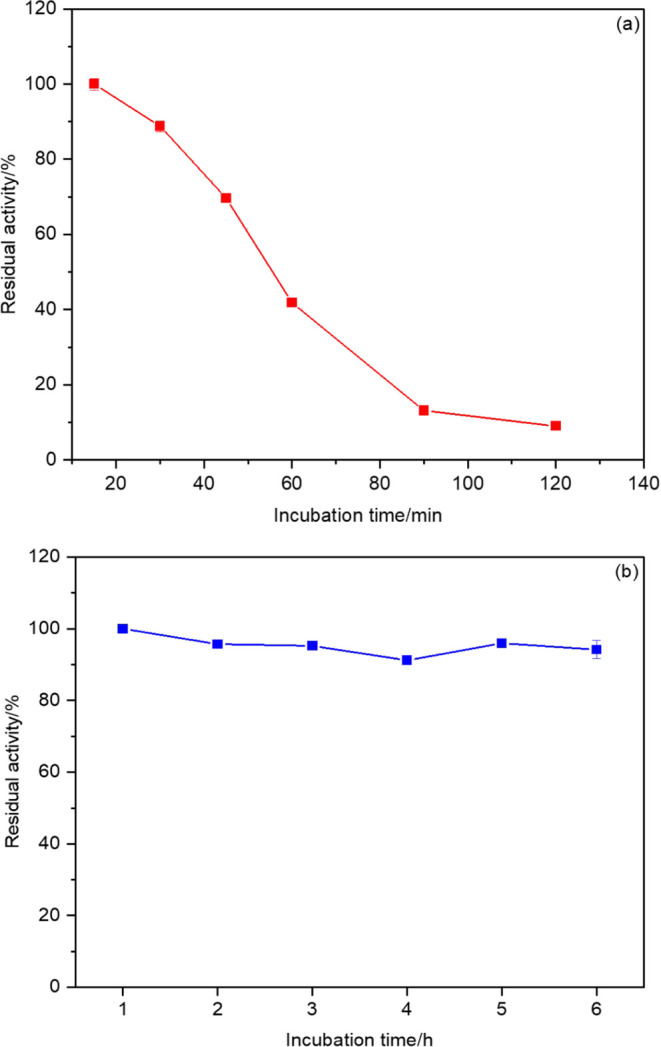
Thermal stability of free HRP (a) and HRP@Fe_3_O_4_–NH_2_/hNFs (b).

Thermal stabilities of free HRP and HRP@Fe_3_O_4_–NH_2_/hNFs are shown in Figure 7. Enzymatic activities in determining the thermal stability of free HRP and HRP@Fe_3_O_4_–NH_2_/hNFs were carried out under the optimum conditions. The thermal stability of free HRP was determined by incubating it at 65 °C for different times (15, 30, 45, 60, 90, and 120 min). Free HRP could only maintain 10% of its activity after 120 min. The HRP@Fe_3_O_4_–NH_2_/hNFs retained 94% of their activity by incubating at 80 °C for 6 h. Thermal stability of the HRP@Fe_3_O_4_–NH_2_/hNFs exhibited highly improved thermostability compared to that of literature studies. For example, the residual activity of immobilized HRP on graphene oxide/magnetic chitosan beads remained below 40% after 2 h of incubation at 75 °C.^65^ In another study, HRP was immobilized to phospholipid-templated titania particles and retained 62.21% of its initial activity after 5 h of incubation at 60 °C.^66^ This remarkable thermal stability can be attributed to the reduction of protein–protein interactions and the reduced vibration intensity of the enzyme when exposed to high temperatures, the higher protein stiffness caused by immobilization on a support,^61^ and the protection provided by the Fe_3_O_4_–NH_2_/hNFs.

#### Reusability and Storage Stability

**Figure 8:**
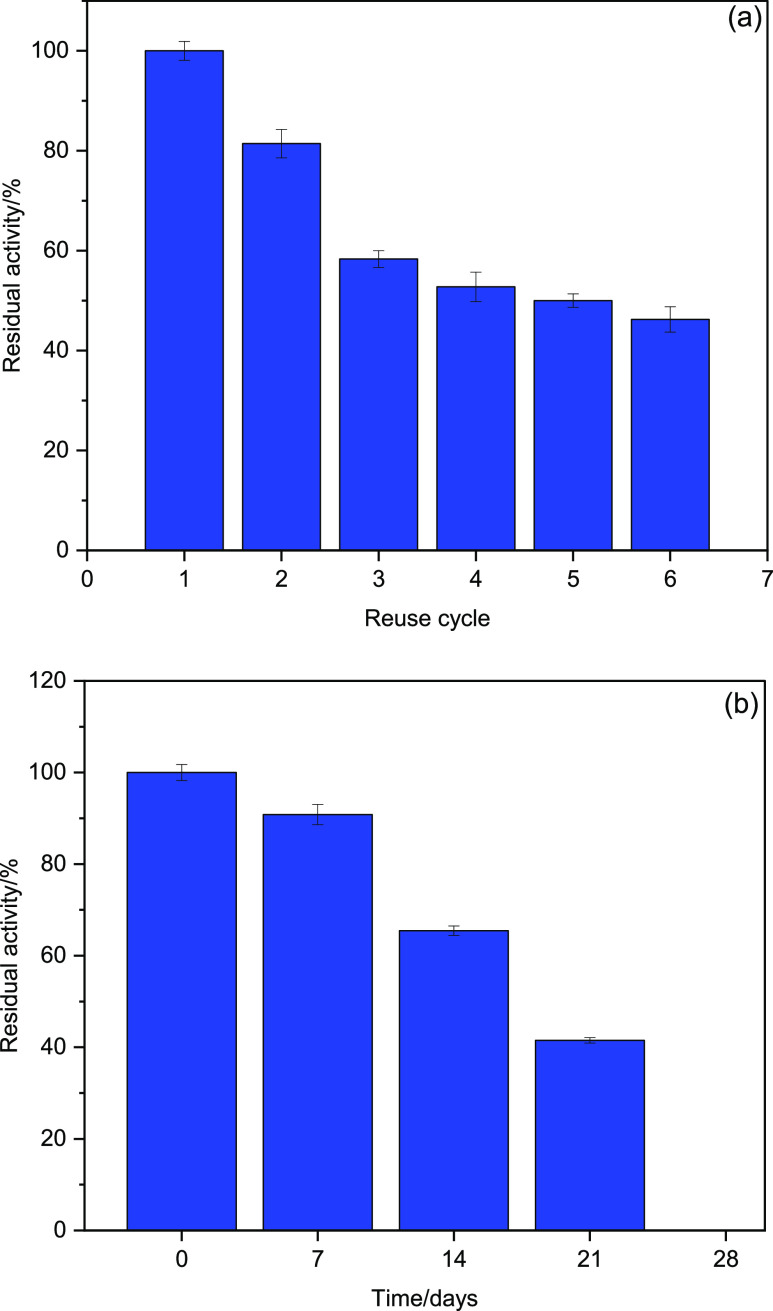
(a) Reuse cycle and (b) storage stability of HRP@Fe_3_O_4_–NH_2_/hNFs.

Reusability is a key parameter in enzyme immobilization. Since the biocatalyst is reusable, the operating costs of biotechnological procedures can be reduced. Therefore, it makes an important economic contribution to industrial processes.^67^ The HRP@Fe_3_O_4_–NH_2_/hNFs were reused for 6 cycles, and the result was displayed in Figure 8a. The residual activities of HRP@Fe_3_O_4_–NH_2_/hNFs in 2 and 4 cycles were 81 and 53%, respectively. The activity of HRP@Fe_3_O_4_–NH_2_/hNFs gradually decreased during the cycles; however, the HRP@Fe_3_O_4_–NH_2_/hNFs retained 46% of its initial activity after 6 cycles of reuse. The loss of activity could be due to possible enzyme leaching from the support. The reusability of HRP was much higher than or equal to that of immobilized counterparts reported in other systems.^68,69^ A comparison was made in terms of the reusability between HRP@Fe_3_O_4_–NH_2_/hNFs and other supports previously reported in the literature (Table 3). Herein, it can be clearly stated that HRP@Fe_3_O_4_–NH_2_/hNFs were better than or comparable to the results obtained from studies using other HRP immobilized materials. Taken together, this study proposes a promising material to degrade textile dyes in wastewater, and the preliminary data obtained are expected to guide further applications of decolorization studies. Moreover, we concluded that HRP@Fe_3_O_4_–NH_2_/hNFs can be prepared on an industrial scale and applied in industrial wastewater treatment processes in the near future.

**Table 3:** Comparison of Reusability of HRP@Fe_3_O_4_–NH_2_/hNFs with Previously Reported Studies

support	reuse cycle	residual Activity (%)	references
HRP-CuO %1/PMMA	10	52	(68)
NH_2_-MOF-Zr@HRP	10	59	(70)
2% Fe_3_O_4_Np–PMMA	10	approximately 15	(71)
HRP-NF	10	50	(72)
Fe_3_O_4_@NH_2_–HRP	8	approximately 20	(21)
H-MNFs	5	52	(73)
HRP@Fe_3_O_4_–NH_2_/hNFs	6	46	this study

Similar to reusability, the storage stability of a biocatalyst is another important parameter of the reliability of the biocatalytic material.^74^ The storage stability of HRP@Fe_3_O_4_–NH_2_/hNFs was checked at 25 °C for 21 days. As seen in Figure 8b, HRP@Fe_3_O_4_–NH_2_/hNFs retained 65.45% of its initial activity at the end of 14 days and 41.50% at the end of 21 days. The remarkable storage stability of HRP@Fe_3_O_4_–NH_2_/hNFs can be attributed to the formation of stable structures with the enzyme as well as to the possible protective effects of the microenvironment around the enzyme molecules. Therefore, the active sites and general configuration of HRP molecules may not have been degraded under storage conditions.^74^

#### Organic Solvents Stability

The activity of the enzyme can be affected by the hydrophobicity or polarity of the solvent. In Figure S5, the activity of the HRP@Fe_3_O_4_–NH_2_/hNFs experienced a decrease when the solvents were added. While approximately 65% of HRP activity was inhibited by tetrahydrofuran (THF) and dimethyl sulfoxide (DMSO), it retained more than 50% of its activity in the presence of dichloromethane (DCM), *n*-hexane, and dimethylformamide (DMF). This may be due to inactivation by disruption of the nonpolar or polar environment or due to reduced accessibility of the substrate to the active sites of the enzyme.^75^ Consequently, activity in the presence of various organic solvents may increase the potential for the industrial use of HRP.

#### Enzymatic Decolorization of Textile Dyes

**Figure 9:**
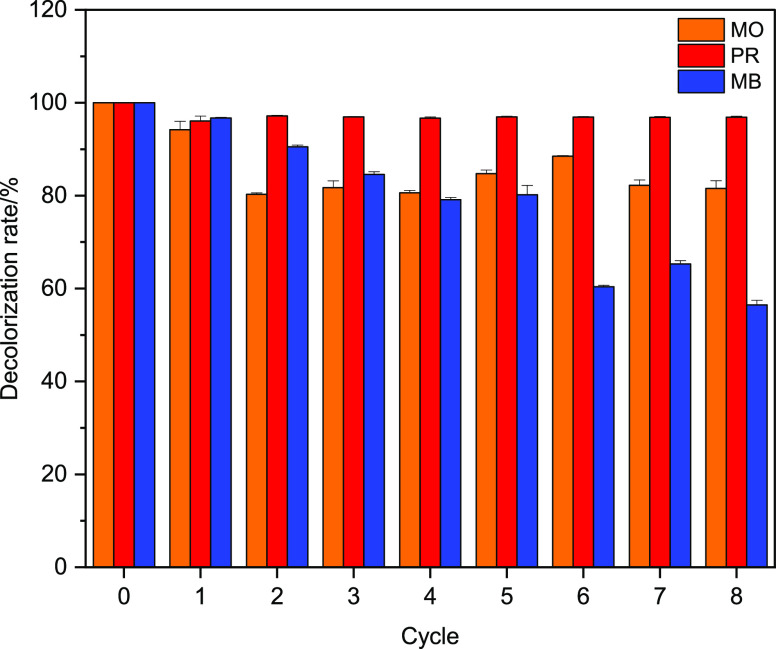
Cyclic decolorization of MO, PR, and MB by HRP@Fe_3_O_4_–NH_2_/hNFs at pH 8.0 and 80 °C.

**Figure 10:**
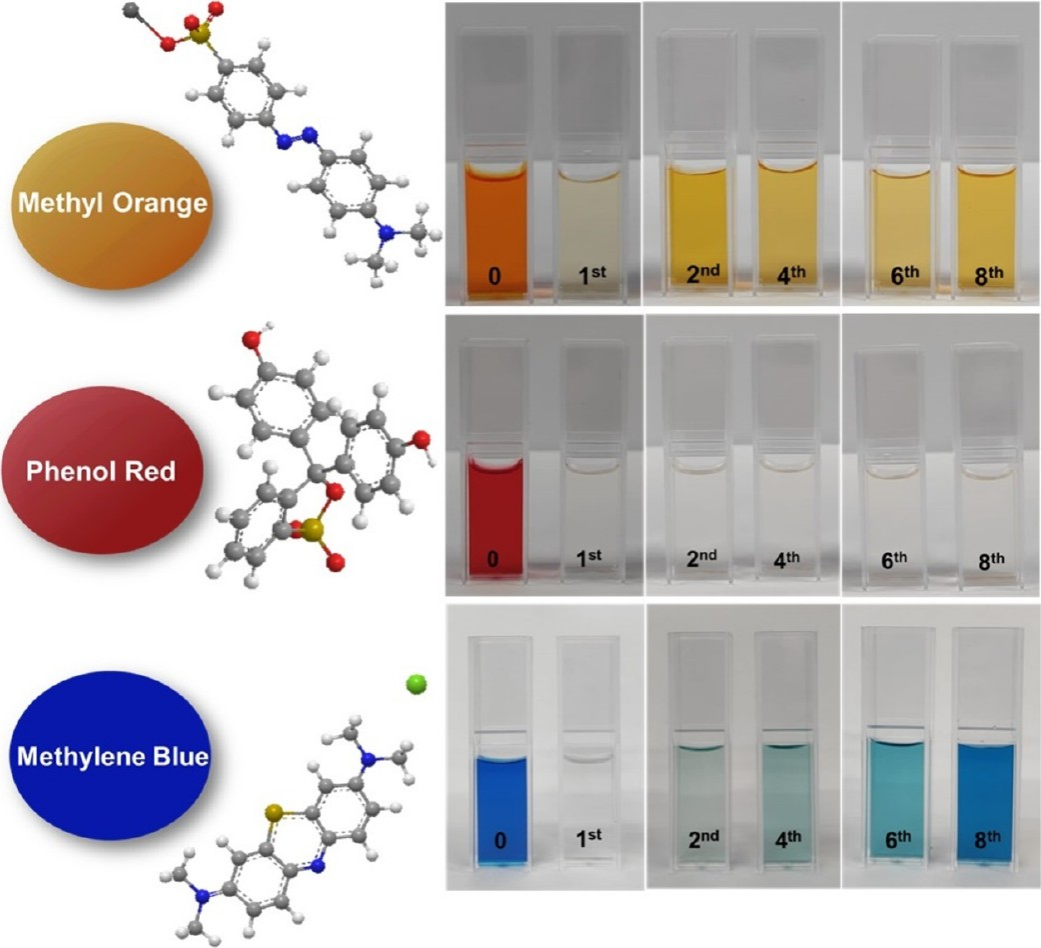
Comparative dye decolorization by HRP@Fe_3_O_4_–NH_2_/hNFs after eight cycles.

The HRP@Fe_3_O_4_–NH_2_/hNFs were applied to decolorize three textile dyes including MO, PR, and MB. The efficiency of enzymatic degradation of dye solutions (150 mg/L) by HRP@Fe_3_O_4_–NH_2_/hNFs at pH 8.0 and 80 °C was investigated. The experimental results are listed in Figure 9. Also, Figure 10 shows comparative dye decolorization by HRP@Fe_3_O_4_–NH_2_/hNFs after the eighth cycle. The results indicated that HRP@Fe_3_O_4_–NH_2_/hNFs were able to highly decolorize both dyes for several cycles. The HRP@Fe_3_O_4_–NH_2_/hNFs exhibited 81% degradation for MO, 96% degradation for PR, and 56% degradation for MB after 8 consecutive cycles. The low decrease of enzyme activity in successive decolorization cycles may be explained by the blocking of some pores of the HRP@Fe_3_O_4_–NH_2_/hNFs by dye and/or degradation products after repeated use.^76^ This has also been reported for other immobilized enzymes used in the decolorization of dyes. On the other hand, differences in the rate of decolorization shown by HRP@Fe_3_O_4_–NH_2_/hNFs for the three dyes depend on the molecular structures of the dyes and/or reaction products.^76^ The decolorization activity of HRP@Fe_3_O_4_–NH_2_/hNFs was much higher than or equal to that of their immobilized counterparts reported in other systems. For instance, Weber et al.^61^ reported that immobilized HRP on calcium alginate-starch hybrid support achieved around 30% color elimination from a solution of 100 mg/L of PR after 8 cycles. In another work, HRP isolated from fresh leaves of *Moringa Oliefera* on iron oxide NPs degraded reactive blue 221 and direct blue 297 dyes by 0.6 and 12%, respectively, after the fifth cycle.^60^ Based on the result obtained in the present study, HRP@Fe_3_O_4_–NH_2_/hNFs show great promise in environmental applications, including wastewater treatment and enzymatic water purification.

#### Morphological Characterization of HRP@Fe_3_O_4_–NH_2_/hNFs after Dye Decolorization

**Figure 11:**
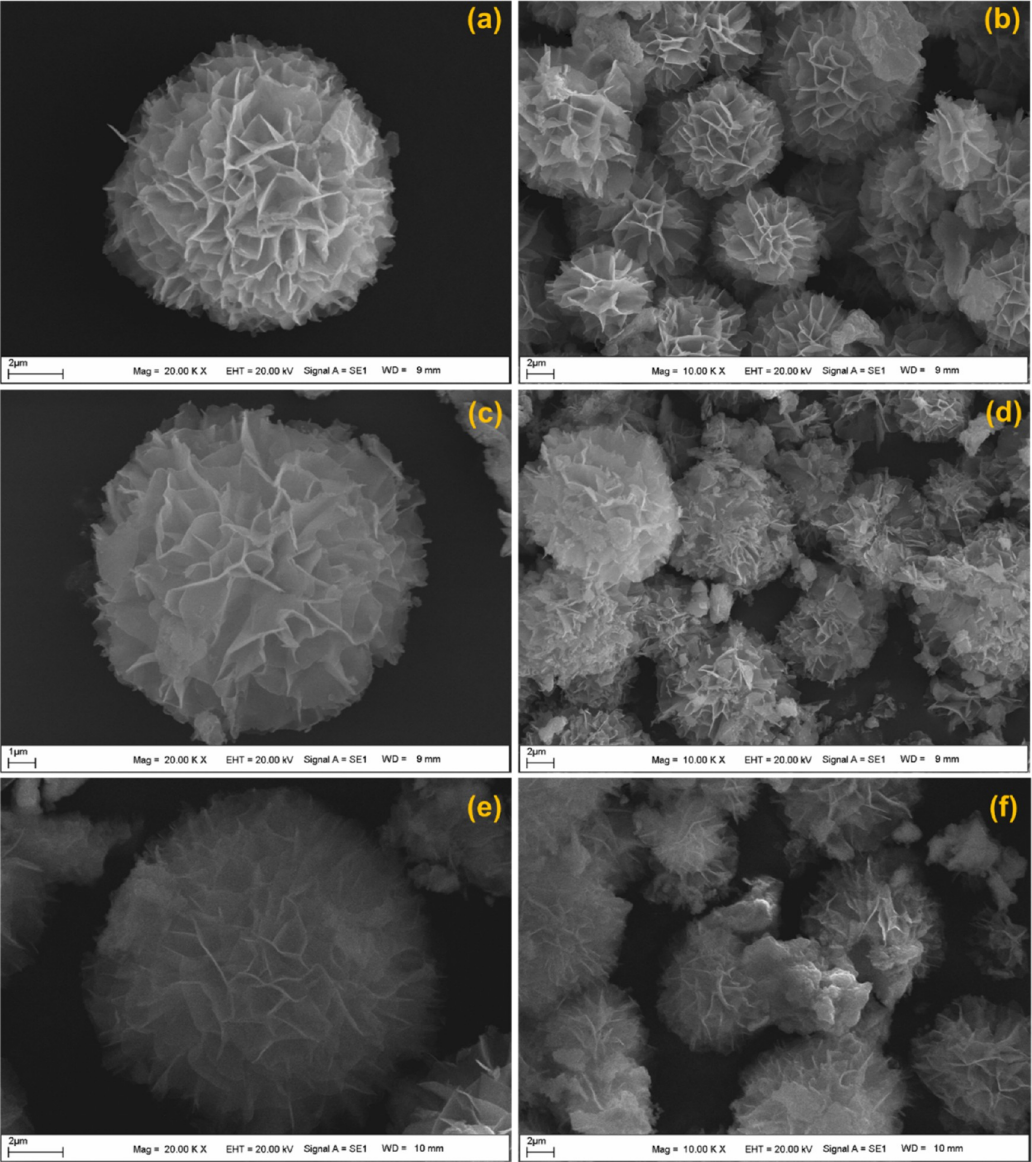
SEM images of the HRP@Fe_3_O_4_–NH_2_/hNFs for MO (a, b), PR (c, d), and MB (e, f) after 8 reuse cycles. (a, c, e): 20.00 K X; (b, d, f): 10.00 K X.

To confirm possible morphological and elemental distribution changes in the HRP@Fe_3_O_4_–NH_2_/hNFs after enzymatic dye decolorization, HRP@Fe_3_O_4_–NH_2_/hNFs subjected to 8 reuse cycles were analyzed via SEM and EDS. Figures 11 and S4 represent the SEM and EDS results, respectively. The flower-like morphology of the HRP@Fe_3_O_4_–NH_2_/hNFs showed no significant structural changes, and the HRP@Fe_3_O_4_–NH_2_/hNFs retained surface properties even after 8 uses. On the other hand, it was clear that some changes had occurred in the elemental composition of the HRP@Fe_3_O_4_–NH_2_/hNFs, as seen in the evaluation by EDS. There was a decrease in C and O contents and the presence of sodium (Na) content, which can be attributed to partial adsorption of dyes or degradation products.

## Conclusions

Briefly speaking, Fe_3_O_4_–NH_2_/hNFs were synthesized using efficient, fast, and low-cost methods and used as the carrier of immobilizing HRP for the first time. As a result of FTIR, XRD, SEM, and EDS characterizations of HRP@Fe_3_O_4_–NH_2_/hNFs, the flower-like morphology of the enzyme-inorganic hNFs and the presence of magnetic NPs incorporated into the hNFs were confirmed. The biochemical parameters of free HRP and HRP@Fe_3_O_4_–NH_2_/hNFs were examined. The optimal pH and temperature of free HRP were determined to be 5.0 and 30 °C, while they were found to be 8.0 and 80 °C for HRP@Fe_3_O_4_–NH_2_/hNFs, respectively. Additionally, the HRP@Fe_3_O_4_–NH_2_/hNFs exhibited significant storage stability (∼40% residual activity) for up to 21 days. Notably, the HRP@Fe_3_O_4_–NH_2_/hNFs displayed outstanding thermal stability and superior reusability compared to free HRP. The residual activity exceeded 45% after 6 cycles and was still ongoing. Meanwhile, the high operational stability of HRP@Fe_3_O_4_–NH_2_/hNFs was crucial for dye decolorization studies. Last but not least, it was observed that HRP@Fe_3_O_4_–NH_2_/hNFs were able to efficiently decolorize around 81% of MO within 8 h. Similarly in the case of PR, a degradation of around 96% was obtained in 8 h. The flower-like morphology of the HRP@Fe_3_O_4_–NH_2_/hNFs remained unchanged after 8 reuse cycles. Overall, the HRP@Fe_3_O_4_–NH_2_/hNFs showed good stability and expanded their application range. These results provided new guiding ideas for the application of HRP@Fe_3_O_4_–NH_2_/hNFs in actual dye wastewater decolorization. Moreover, we anticipated that this research could lead to a new alternative that will benefit the sustainable development of the textile industry and the protection of the environment.

## Materials and Methods

### Materials

Lyophilized peroxidase from horseradish (∼150 U/mg), guaiacol (oxidation indicator), H_2_O_2_ solution (34.5–36.5%), copper sulfate pentahydrate (CuSO_4_·5H_2_O), poly(ethylene glycol) 35000 (PEG 35000), sodium hydroxide (NaOH), tetraethoxysilane (TEOS), 3-aminopropyl triethoxysilane (APTES), hydrochloric acid (HCl), triethylene glycol, urea, ethanol, sodium dihydrogen phosphate monohydrate (NaH_2_PO_4_·H_2_O), sodium chloride (NaCl), potassium chloride (KCl), disodium hydrogen phosphate dihydrate (Na_2_HPO_4_·2H_2_O), potassium dihydrogen phosphate (KH_2_PO_4_), magnesium chloride heptahydrate (MgCl_2_·6H_2_O), calcium chloride dihydrate (CaCl_2_·2H_2_O), bovine serum albumin (BSA), Coomassie Brilliant Blue G-250, and MB (C_16_H_18_ClN_3_S) were obtained from Sigma-Aldrich (St. Louis MO). MO (C_14_H_14_N_3_NaO_3_S) and PR (C_19_H_14_O_5_S) were purchased from Merck (Darmstadt, Germany) and Carlo Erba Chemicals, respectively. Unless stated otherwise, other chemicals were of analytical grade and were used without further purification. Throughout the experiments, substrate and enzyme solutions were prepared fresh with an appropriate buffer solution. Ultrapure water produced by a Millipore Direct-Q3 System was used to prepare the aqueous solutions.

### Instruments

FTIR spectra were performed with a spectrophotometer (PerkinElmer) at 400–4000 cm^–1^. The magnetization value of the HRP@Fe_3_O_4_–NH_2_/hNFs was determined using Quantum Design VSM-Physical Property Measurement System (PPMS) with a magnetic field of −60 000 ≤ Oe ≤ 60 000. XRD pattern was collected via powder XRD (Rigaku RadB-DMAX II) with Cu Kα radiation in the 10–80° of 2θ range. A high-resolution transmission electron microscopy (HR-TEM) image of APTES-coated mesoporous magnetic Fe_3_O_4_–NH_2_ NPs was studied using TEM (Jeol. 2100F RTEM 200 kV). The surface morphology of the HRP@Fe_3_O_4_–NH_2_/hNFs was investigated by using SEM (LEO Evo-40 VPX, Cambridge, U.K.). To identify the elemental composition, EDS (model 125 eV; Bruker AXS, Berlin, Germany) analysis was carried out. A DLS (detection angle 90°, Litesizer 500 Model, Anton Paar, Graz, Austria) was used to determine the hydrodynamic diameter and ζ-potential of the HRP@Fe_3_O_4_–NH_2_/hNFs. Measurements of Brunauer–Emmett–Teller (BET) were carried out using a BET surface area analyzer (Quantachrome Corporation, Autosorb-6).

### Synthesis of Amino-Functionalized Mesoporous Magnetic NPs (Fe_3_O_4_–NH_2_)

**Figure 12:**
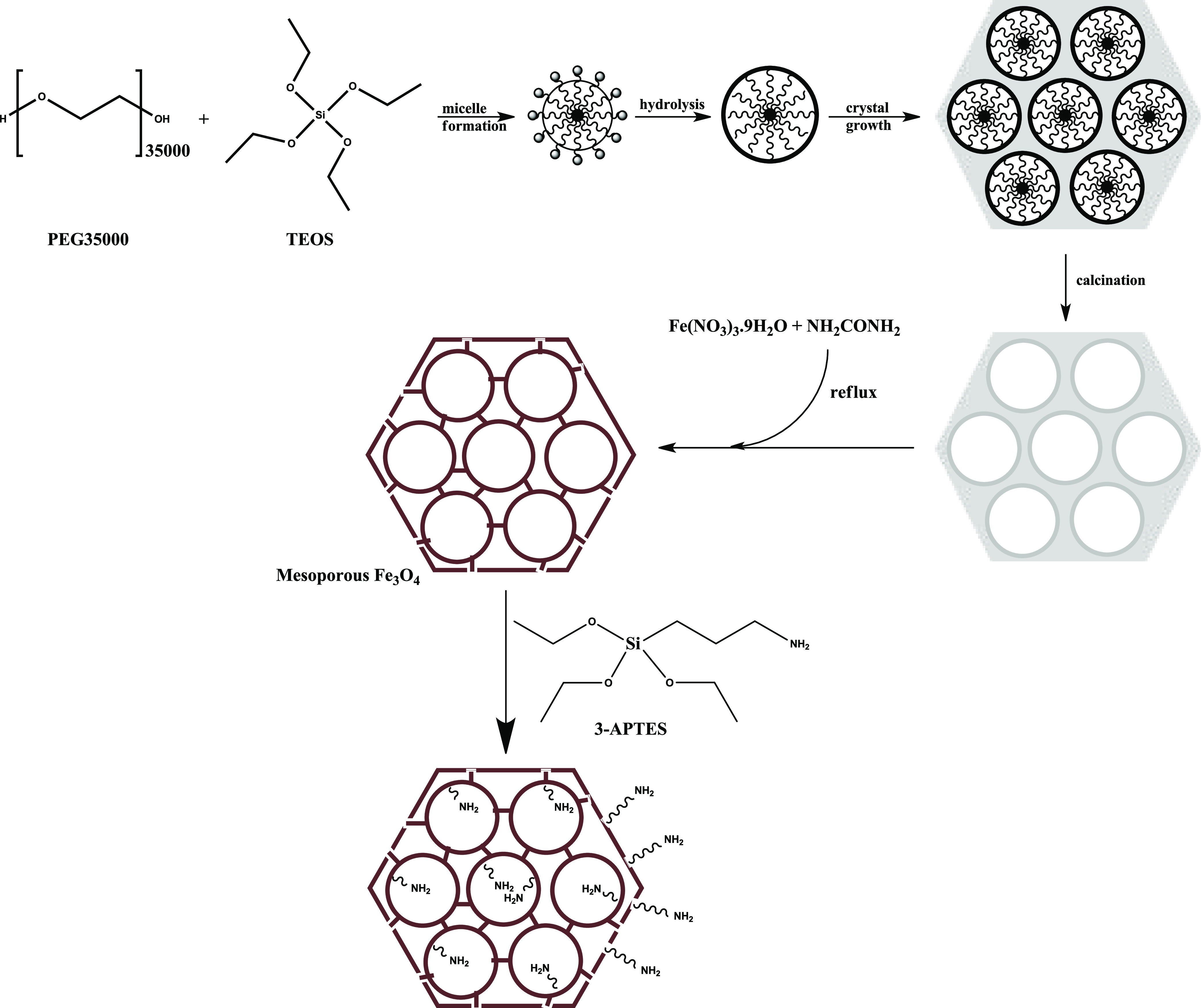
A schematic illustration of the synthesis of mesoporous magnetic Fe_3_O_4_–NH_2_ NPs.

Magnetic mesoporous Fe_3_O_4_ NPs were prepared according to the method developed by Ulusal and Özdemir.^77^ First, mesoporous SiO_2_ was prepared, and its surface was coated with Fe_3_O_4_ using Fe(urea)_6_, and then SiO_2_ was leached to obtain mesoporous Fe_3_O_4_. Approximately 1.5 g of the prepared mesoporous Fe_3_O_4_ compound was taken and mixed in 100 mL of pure acetone in an ultrasonic bath until it was completely homogeneous (approximately 30 min). 4 mL of APTES (4% by volume) was added to this mixture, and the final mixture was magnetically stirred at 45 °C for 24 h. At the end of this period, it was washed with pure water and dried at 115 °C overnight. At the end of the experiment, approximately 1.8 g of product was obtained. The schematic synthesis of the mesoporous magnetic Fe_3_O_4_–NH_2_ NPs is demonstrated in Figure 12.

### Preparation of HRP@Fe_3_O_4_–NH_2_ NPs

**Figure 13:**
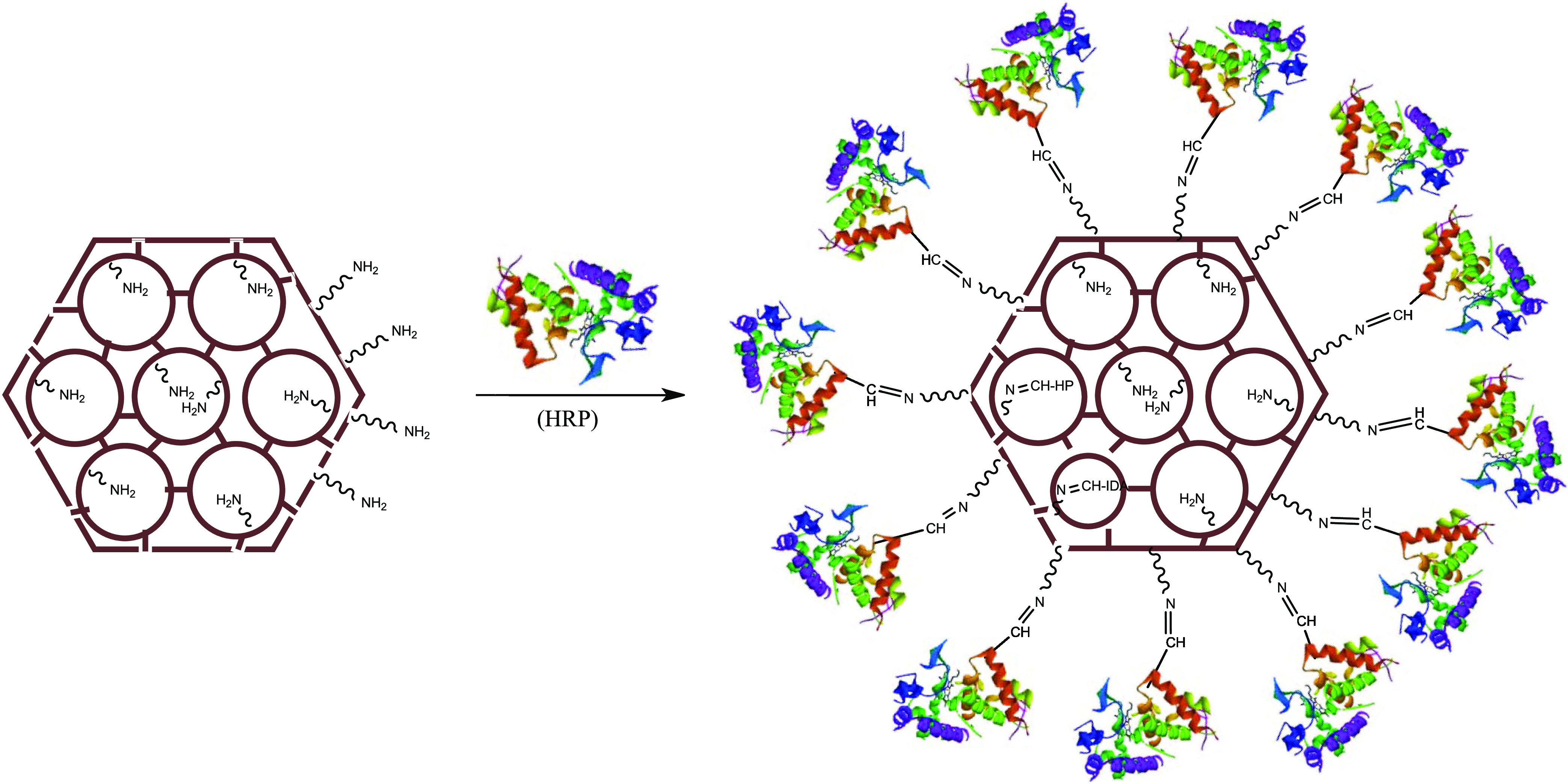
Schematic representation of HRP immobilization on mesoporous magnetic Fe_3_O_4_–NH_2_ NPs.

The procedure for preparing HRP@Fe_3_O_4_–NH_2_ NPs is described in Figure 13. 15 mg of mesoporous magnetic Fe_3_O_4_–NH_2_ NPs were weighed and added to 15 mL of phosphate-buffered saline (PBS, pH 7.4). This mixture was sonicated for 30 min. After that, a certain amount of HRP enzyme was added to this mixture. The final HRP concentration was 0.5 mg/mL in the adsorption medium. This mixture was shaken in a temperature-controlled shaking bath for 3 h. At the end of this period, the HRP-immobilized Fe_3_O_4_–NH_2_ NPs were separated from the supernatant by using a magnet. The amount of HRP remaining in the supernatant (not immobilized on Fe_3_O_4_–NH_2_) was determined with the Bradford protein assay.^78^

### Synthesis of HRP@Fe_3_O_4_–NH_2_/hNFs

The synthesis of magnetic enzyme-inorganic hybrid nanoflowers (HRP@Fe_3_O_4_–NH_2_/hNFs) was carried out following a recently published procedure.^29,79^ Just like in the synthesis of nonmagnetic enzyme-inorganic hybrid nanoflowers, 3 days is sufficient time for the synthesis of HRP@Fe_3_O_4_–NH_2_/hNFs. Similarly, just like the synthesis of nonmagnetic enzyme-inorganic hNFs, the synthesis of magnetic HRP@Fe_3_O_4_–NH_2_/hNFs can be explained in three steps (nucleation, growth, and completion).

#### I Nucleation Step

Primary copper phosphate nanocrystals occur. While copper phosphate nanocrystals form in the early stage, the amino moieties located on the surface of Fe_3_O_4_–NH_2_ NPs and HRP interact with the Cu(II) ions in the copper phosphate and form complexes.

#### II Growth Step

With an increasing incubation time, large agglomerates are formed through the coordination interaction between HRP@Fe_3_O_4_–NH_2_ NPs and Cu(II) ions, which will then form HRP@Fe_3_O_4_–NH_2_/hNFs.

#### III Completion Step

After 3 days of incubation, flower-like hierarchical structures (HRP@Fe_3_O_4_–NH_2_/hNFs) with a high surface/volume ratio are formed in which HRP@Fe_3_O_4_–NH_2_ NPs are embedded.

**Figure 14:**
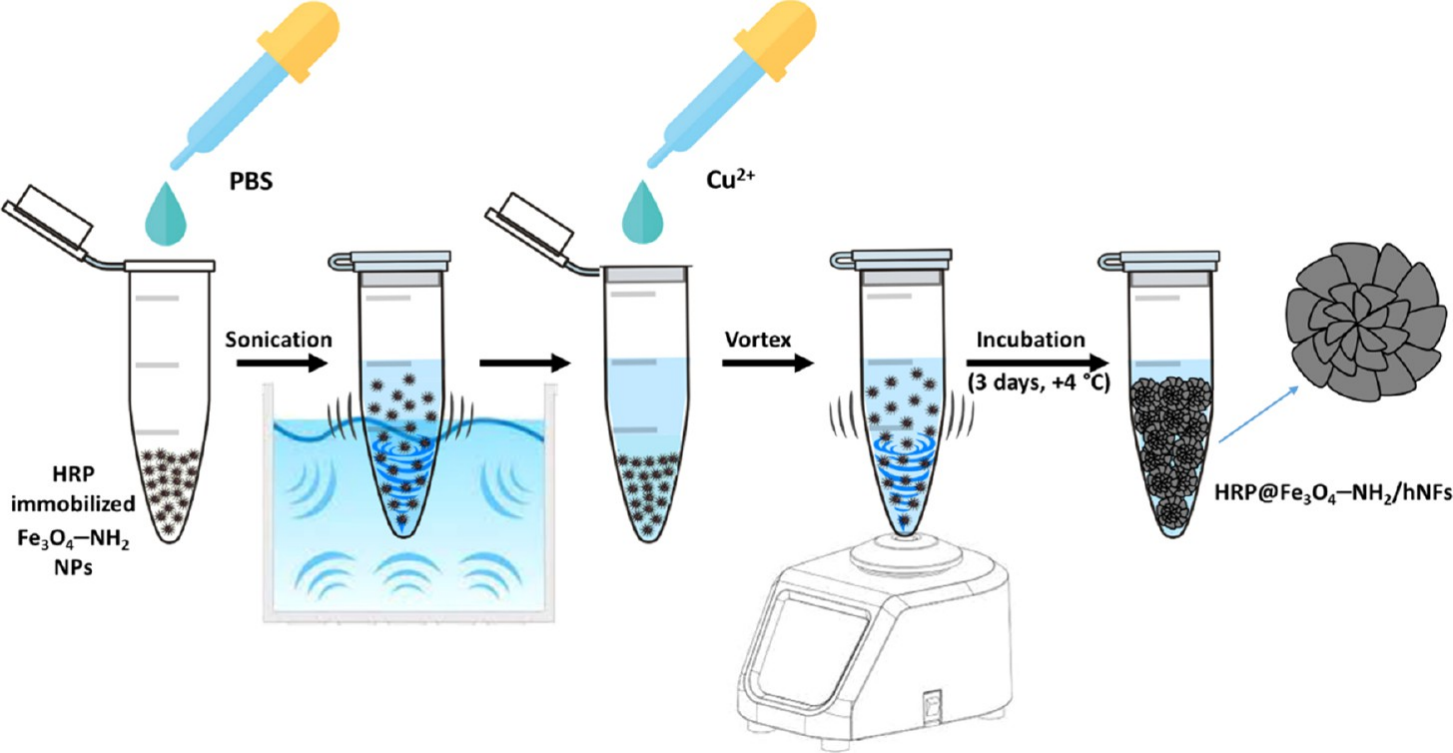
Schematic representation of the synthesis of HRP@Fe_3_O_4_–NH_2_/hNFs.

For this purpose, the HRP@Fe_3_O_4_–NH_2_ NPs prepared in the previous step were dispersed in PBS and this mixture was sonicated for a while. Then a certain amount of Cu(II) ion solution was added, and this mixture was vortexed quickly. At the end of all of these processes, this mixture was incubated for 3 days. After 3 days, it was observed that a gray precipitate (indicating the formation of HRP@Fe_3_O_4_–NH_2_/hNFs) occurred. This precipitate was separated from the solution by using a magnet, washed at least three times with pure water, and left to dry. A schematic illustration of the synthesis of HRP@Fe_3_O_4_–NH_2_/hNFs is given in Figure 14.

### Enzyme Activity Assay

HRP activity was determined by measuring the oxidation of guaiacol to tetraguaiacol in the presence of H_2_O_2_ spectrophotometrically at 470 nm.^21^ 40 mM guaiacol and 40 mM H_2_O_2_ solutions were prepared in phosphate buffer (50 mM, pH 7.0). 0.02 U of HRP and 1.5 mg of HRP@Fe_3_O_4_–NH_2_/hNFs were incubated in a 1 mL reaction mixture consisting of 500 μL of guaiacol (40 mM) and 500 μL of H_2_O_2_ (40 mM) for 15 min at 30 °C. Then, the HRP@Fe_3_O_4_–NH_2_/hNFs were centrifuged at 5000 rpm for 5 min, and the absorbance of tetraguaiacol due to enzymatic oxidation of guaiacol was monitored at 470 nm using the Gen5 Data Analysis software interface microplate reader (Bio-Tek Instruments, Winooski). One unit of peroxidase activity was determined as the amount of enzyme capable of catalyzing the oxidation of 1 μmol of guaiacol per minute. In all conditions, the measurements were made in triplicate, and data were presented as mean ± standard deviation (SD).

### Biochemical Characterization

#### Calculation of Immobilization Yield

Immobilization yield was calculated using eq 1:
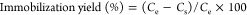
1where *C*_s_ is the protein concentration in the supernatant and *C*_e_ is the protein concentration of the HRP used for immobilization.

#### Effect of pH and Temperature

The effect of changing pH on the activity of free HRP and HRP@Fe_3_O_4_–NH_2_/hNFs was examined using acetate buffer (pH 4.0, 5.0, and 6.0), phosphate buffer (pH 7.0 and 8.0), and Tris-HCl buffer (pH 9.0 and 10.0). The effect of changing reaction temperatures on the activity of HRP and HRP@Fe_3_O_4_–NH_2_/hNFs was determined by measuring at 20–90 °C for free HRP and HRP@Fe_3_O_4_–NH_2_/hNFs. The activity at optimum conditions of pH and temperature was accepted as 100% and the results were given as the relative activity. The pH and temperature values with the highest activity were determined as optimum.

Additionally, the logarithm of % relative activity versus 1000/*T* was plotted, and the activation energy (*E*_a_) of free HRP and HRP@Fe_3_O_4_–NH_2_/hNFs was calculated from the slope of the Arrhenius plot using eq 2.
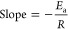
2Here, *E*_a_ is the activation energy (kJ/mol) and *R* is the gas constant = 8.314 J/(mol K).

#### Determination of Kinetic Parameters

The kinetic parameters (*K*_m_ and *V*_max_) of free HRP and HRP@Fe_3_O_4_–NH_2_/hNFs were examined under optimum conditions by varying the H_2_O_2_ concentration between 5 and 20 mM when the guaiacol concentration (20 mM) concentration was constant. *K*_m_ and *V*_max_ values were calculated with Lineweaver–Burk plots.

#### Thermal Stability

Thermal stability of free HRP was determined by incubating 0.01 U of HRP enzyme for 15, 30, 45, 60, 90, and 120 min at a reaction temperature of 65 °C, which is well above its optimum temperature. The activity of free HRP after 15 min was accepted as 100% and the results were given as the relative activity. The thermal stability of HRP@Fe_3_O_4_–NH_2_/hNFs was determined by taking measurements at 1 h intervals during 6 h of incubation at 80 °C. The activity of HRP@Fe_3_O_4_–NH_2_/hNFs after 1 h was considered 100%, and the results were presented as relative activity.

#### Reusability and Storage Stability

The reusability of HRP@Fe_3_O_4_–NH_2_/hNFs was examined by successively measuring its activity under optimum conditions (pH 8.0 and 80 °C). After each activity measurement, HRP@Fe_3_O_4_–NH_2_/hNFs were separated from the reaction medium by centrifugation and washed with 1 mL of distilled water, and activity measurement was performed again. These steps were repeated for 6 cycles. The activity measured after each repetition was determined by accepting the initial activity as 100%.

The storage stability of the HRP@Fe_3_O_4_–NH_2_/hNFs was measured by keeping them at 25 °C for 21 days. The enzyme activity determined on the first day was accepted as 100% and the following days were presented as relative activity.

#### Organic Solvents Stability

The solvent stability of HRP@Fe_3_O_4_–NH_2_/hNFs was also determined in the presence of various organic solvents dichloromethane (DCM), *n*-hexane, dimethylformamide (DMF), tetrahydrofuran (THF), and dimethyl sulfoxide (DMSO) under the assay reaction conditions. The solvent stability of the HRP@Fe_3_O_4_–NH_2_/hNFs was studied by incubating the enzyme in 5% organic solvent for 24 h. The activity without organic solvent was taken as 100%.

#### Enzymatic Decolorization of Textile Dyes

Three different model dyes were used for enzymatic decolorization of the textile dye. The chemical structures of MO (anionic azo dye), PR (trimethylmethane dye), and MB (cationic thiazine dye) are shown in Figure S5. The enzymatic biodegradation of MO and PR dyes was conducted in dedicated flasks, which is similar to that found in the optimum reaction conditions of the HRP@Fe_3_O_4_–NH_2_/hNFs, according to the method described by Bilal et al.^80^ A mass of 5.0 mg of the HRP@Fe_3_O_4_–NH_2_/hNFs was incubated with 3.0 mL of dye solution (150 mg/L) in 50 mM phosphate buffer (pH 8.0) at 80 °C, and the reaction was initiated by 0.6 mL of H_2_O_2_ with 100 mM with continuous magnetic stirring. After each cycle, the derivative was separated, washed with ultrapure water, and added to a fresh dye solution for the subsequent cycle. This process was repeated for 8 cycles and at the end of each cycle, an aliquot of the supernatant was collected for the determination of the decolorization of dye solutions. The decolorization percentage of the supernatants containing MO (at 465 nm), PR (at 430 nm), and MB (665 nm) was measured by a microplate reader (Bio-Tek Instruments, Winooski). All experiments were performed in triplicate, and the results were expressed as mean ± SD values. The enzymatic decolorization every hour after applying the HRP@Fe_3_O_4_–NH_2_/hNFs was calculated using eq 3:
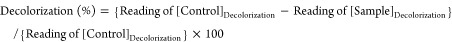
3The decolorization observed in the first cycle was defined as 100% and the following measurements were calculated as relative percentages. Control experiments were also carried out under the same reaction conditions but in the absence of HRP@Fe_3_O_4_–NH_2_/hNFs.

## Supporting Information Available

BET, HR-TEM, EDS data of the materials, and chemical structures of dyes (PDF)

## References

[ref1] Calza P.; Zacchigna D.; Laurenti E. Degradation of orange dyes and carbamazepine by soybean peroxidase immobilized on silica monoliths and titanium dioxide. Environ. Sci. Pollut. Res. 2016, 23 (23), 23742–23749. 10.1007/s11356-016-7399-1. 27623850

[ref2] Khalid N.; Kalsoom U.; Ahsan Z.; Bilal M. Non-magnetic and magnetically responsive support materials immobilized peroxidases for biocatalytic degradation of emerging dye pollutants-A Review. Int. J. Biol. Macromol. 2022, 207, 387–401. 10.1016/j.ijbiomac.2022.03.035. 35278508

[ref3] Al-Tohamy R.; Ali S. S.; Li F.; Okasha K. M.; Mahmoud Y. A. G.; Elsamahy T.; Jiao H.; Fu Y.; Sun J. A critical review on the treatment of dye-containing wastewater: ecotoxicological and health concerns of textile dyes and possible remediation approaches for environmental safety. Ecotoxicol. Environ. Saf. 2022, 231, 113160 10.1016/j.ecoenv.2021.113160. 35026583

[ref4] Acharya R.; Naik B.; Parida K. M. Adsorption of Cr (VI) and Textile Dyes on to Mesoporous Silica, Titanate Nanotubes, and Layered Double Hydroxides. In Nanomaterials in the Wet Processing of Textiles; Wiley, 2018; pp 219–260.

[ref5] Zdarta J.; Jankowska K.; Bachosz K.; Degórska O.; Kaźmierczak K.; Nguyen L. N.; Nghiem L. D.; Jesionowski T. Enhanced wastewater treatment by immobilized enzymes. Curr. Pollut. Rep. 2021, 7 (2), 167–179. 10.1007/s40726-021-00183-7.

[ref6] Al-Maqdi K. A.; Elmerhi N.; Athamneh K.; Bilal M.; Alzamly A.; Ashraf S. S.; Shah I. Challenges and recent advances in enzyme-mediated wastewater remediation-a review. Nanomaterials 2021, 11 (11), 3124 10.3390/nano11113124. 34835887 PMC8625148

[ref7] Ariaeenejad S.; Motamedi E.; Hosseini Salekdeh G. Application of the immobilized enzyme on magnetic graphene oxide nano-carrier as a versatile bi-functional tool for efficient removal of dye from water. Bioresour. Technol. 2021, 319, 124228 10.1016/j.biortech.2020.124228. 33254455

[ref8] Bilal M.; Qamar S. A.; Carballares D.; Berenguer-Murcia Á.; Fernandez-Lafuente R. Proteases immobilized on nanomaterials for biocatalytic, environmental and biomedical applications: advantages and drawbacks. Biotechnol. Adv. 2024, 70, 108304 10.1016/j.biotechadv.2023.108304. 38135131

[ref9] Bolivar J. M.; Woodley J. M.; Fernandez-Lafuente R. Is enzyme immobilization a mature discipline? Some critical considerations to capitalize on the benefits of immobilization. Chem. Soc. Rev. 2022, 51 (15), 6251–6290. 10.1039/D2CS00083K. 35838107

[ref10] Boudrant J.; Woodley J. M.; Fernandez-Lafuente R. Parameters necessary to define an immobilized enzyme preparation. Process Biochem. 2020, 90, 66–80. 10.1016/j.procbio.2019.11.026.

[ref11] Dik G.; Bakar B.; Ulu A.; Ateş B. Propelling of enzyme activity by using different triggering strategies: applications and perspectives. Ind. Eng. Chem. Res. 2023, 62 (36), 14111–14129. 10.1021/acs.iecr.3c01678.

[ref12] Barbosa O.; Ortiz C.; Berenguer-Murcia Á.; Torres R.; Rodrigues R. C.; Fernandez-Lafuente R. Strategies for the one-step immobilization–purification of enzymes as industrial biocatalysts. Biotechnol. Adv. 2015, 33 (5), 435–456. 10.1016/j.biotechadv.2015.03.006. 25777494

[ref13] Saikia S.; Gogoi R. D.; Yadav M.; Yadav H. S. Isolation, purification and characterization of peroxidase from raphanus sativus and its applications in biotransformation of cresols. Biocatal. Agric Biotechnol. 2022, 46, 102540 10.1016/j.bcab.2022.102540.

[ref14] Sellami K.; Couvert A.; Nasrallah N.; Maachi R.; Abouseoud M.; Amrane A. Peroxidase enzymes as green catalysts for bioremediation and biotechnological applications: a review. Sci. Total Environ. 2022, 806, 150500 10.1016/j.scitotenv.2021.150500. 34852426

[ref15] Battistuzzi G.; Bellei M.; Bortolotti C. A.; Sola M. Redox properties of heme peroxidases. Arch. Biochem. Biophys. 2010, 500 (1), 21–36. 10.1016/j.abb.2010.03.002. 20211593

[ref16] Abdulwahhab Mohammed W.; M-Ridha M. J. Extraction and purification techniques of the bio-catalyst cabbage peroxidase enzyme to remove reactive dyes and bisphenol-a pollutants. Results Eng. 2024, 21, 101961 10.1016/j.rineng.2024.101961.

[ref17] Guo C.; Chadwick R. J.; Foulis A.; Bedendi G.; Lubskyy A.; Rodriguez K. J.; Pellizzoni M. M.; Milton R. D.; Beveridge R.; Bruns N. Peroxidase activity of myoglobin variants reconstituted with artificial cofactors. Chem. Bio. Chem. 2022, 23 (18), e202200197 10.1002/cbic.202200197. PMC9545363 35816250

[ref18] Melo M. N.; Pereira F. M.; Rocha M. A.; Ribeiro J. G.; Diz F. M.; Monteiro W. F.; Ligabue R. A.; Severino P.; Fricks A. T. Immobilization and characterization of horseradish peroxidase into chitosan and chitosan/peg nanoparticles: a comparative study. Process Biochem. 2020, 98, 160–171. 10.1016/j.procbio.2020.08.007.

[ref19] Zaak H.; Fernandez-Lopez L.; Otero C.; Sassi M.; Fernandez-Lafuente R. Improved stability of immobilized lipases via modification with polyethylenimine and glutaraldehyde. Enzyme Microb. Technol. 2017, 106, 67–74. 10.1016/j.enzmictec.2017.07.001. 28859812

[ref20] Rodrigues R. C.; Ortiz C.; Berenguer-Murcia Á.; Torres R.; Fernández-Lafuente R. Modifying enzyme activity and selectivity by immobilization. Chem. Soc. Rev. 2013, 42 (15), 6290–6307. 10.1039/C2CS35231A. 23059445

[ref21] Keshta B. E.; Gemeay A. H.; Khamis A. A. Impacts of horseradish peroxidase immobilization onto functionalized superparamagnetic iron oxide nanoparticles as a biocatalyst for dye degradation. Environ. Sci. Pollut. Res. 2022, 29 (5), 6633–6645. 10.1007/s11356-021-16119-z. 34455562

[ref22] Mehta J.; Bhardwaj N.; Bhardwaj S. K.; Kim K.-H.; Deep A. Recent advances in enzyme immobilization techniques: metal-organic frameworks as novel substrates. Coord. Chem. Rev. 2016, 322, 30–40. 10.1016/j.ccr.2016.05.007.

[ref23] Liu D. M.; Dong C. Recent advances in nano-carrier immobilized enzymes and their applications. Process Biochem. 2020, 92, 464–475. 10.1016/j.procbio.2020.02.005.

[ref24] Meena J.; Gupta A.; Ahuja R.; Singh M.; Panda A. K. Recent advances in nano-engineered approaches used for enzyme immobilization with enhanced activity. J. Mol. Liq. 2021, 338, 116602 10.1016/j.molliq.2021.116602.

[ref25] Urrea D. A. M.; Gimenez A. V. F.; Rodriguez Y. E.; Contreras E. M. Immobilization of horseradish peroxidase in ca-alginate beads: evaluation of the enzyme leakage on the overall removal of an azo-dye and mathematical modeling. Process Saf. Environ. Prot. 2021, 156, 134–143. 10.1016/j.psep.2021.10.006.

[ref26] Kurtuldu A.; Eşgin H.; Yetim N. K.; Semerci F. Immobilization horseradish peroxidase onto UiO-66-NH_2_ for biodegradation of organic dyes. J. Inorg. Organomet. Polym. Mater. 2022, 32 (8), 2901–2909. 10.1007/s10904-022-02310-3.

[ref27] Darwesh O. M.; Matter I. A.; Eida M. F. Development of peroxidase enzyme immobilized magnetic nanoparticles for bioremediation of textile wastewater dye. J. Environ. Chem. Eng. 2019, 7 (1), 102805 10.1016/j.jece.2018.11.049.

[ref28] Besharati Vineh M.; Saboury A. A.; Poostchi A. A.; Rashidi A. M.; Parivar K. Stability and activity improvement of horseradish peroxidase by covalent immobilization on functionalized reduced graphene oxide and biodegradation of high phenol concentration. Int. J. Biol. Macromol. 2018, 106, 1314–1322. 10.1016/j.ijbiomac.2017.08.133. 28851646

[ref29] Alhayali N. I.; Kalaycioğlu Özpozan N.; Dayan S.; Özdemir N.; Yılmaz B. S. Catalase/Fe_3_O_4_@Cu^2+^ hybrid biocatalytic nanoflowers fabrication and efficiency in the reduction of organic pollutants. Polyhedron 2021, 194, 114888 10.1016/j.poly.2020.114888.

[ref30] Jonović M.; Jugović B.; Žuža M.; Đorđević V.; Milašinović N.; Bugarski B.; Knežević-Jugović Z. Immobilization of horseradish peroxidase on magnetite-alginate beads to enable effective strong binding and enzyme recycling during anthraquinone dyes’ degradation. Polymers 2022, 14 (13), 2614 10.3390/polym14132614. 35808660 PMC9269335

[ref31] Varamini M.; Zamani H.; Hamedani H.; Namdari S.; Rastegari B. Immobilization of horseradish peroxidase on lysine-functionalized gum arabic-coated Fe_3_O_4_ nanoparticles for cholesterol determination. Prep. Biochem. Biotechnol. 2022, 52 (7), 737–747. 10.1080/10826068.2021.1992780. 34871533

[ref32] Noma S. A. A.; Yılmaz B. S.; Ulu A.; Özdemir N.; Ateş B. Development of L-asparaginase@hybrid nanoflowers (ASNase@HNFs) reactor system with enhanced enzymatic reusability and stability. Catal. Lett. 2021, 151 (4), 1191–1201. 10.1007/s10562-020-03362-1.

[ref33] Xu K.; Appiah B.; Zhang B.-W.; Yang Z.-H.; Quan C. Recent advances in enzyme immobilization based on nanoflowers. J. Catal. 2023, 418, 31–39. 10.1016/j.jcat.2023.01.001.

[ref34] Jafari-Nodoushan H.; Mojtabavi S.; Faramarzi M. A.; Samadi N. Organic-inorganic hybrid nanoflowers: the known, the unknown, and the future. Adv. Colloid Interface Sci. 2022, 309, 102780 10.1016/j.cis.2022.102780. 36182695

[ref35] Patel S. K. S.; Otari S. V.; Li J.; Kim D. R.; Kim S. C.; Cho B.-K.; Kalia V. C.; Kang Y. C.; Lee J.-K. Synthesis of cross-linked protein-metal hybrid nanoflowers and its application in repeated batch decolorization of synthetic dyes. J. Hazard. Mater. 2018, 347, 442–450. 10.1016/j.jhazmat.2018.01.003. 29353189

[ref36] Yu J.; Chen X.; Jiang M.; Wang A.; Yang L.; Pei X.; Zhang P.; Wu S. G. Efficient promiscuous knoevenagel condensation catalyzed by papain confined in Cu_3_(PO_4_)_2_ nanoflowers. RSC Adv. 2018, 8 (5), 2357–2364. 10.1039/C7RA12940H. 35541490 PMC9077389

[ref37] Dube S.; Rawtani D. Understanding intricacies of bioinspired organic-inorganic hybrid nanoflowers: a quest to achieve enhanced biomolecules immobilization for biocatalytic, biosensing and bioremediation applications. Adv. Colloid Interface Sci. 2021, 295, 102484 10.1016/j.cis.2021.102484. 34358991

[ref38] Fu S.; Wang S.; Zhang X.; Qi A.; Liu Z.; Yu X.; Chen C.; Li L. Structural effect of Fe_3_O_4_ nanoparticles on peroxidase-like activity for cancer therapy. Colloids Surf. B Biointerfaces 2017, 154, 239–245. 10.1016/j.colsurfb.2017.03.038. 28347945

[ref39] Patil P. D.; Kelkar R. K.; Patil N. P.; Pise P. V.; Patil S. P.; Patil A. S.; Kulkarni N. S.; Tiwari M. S.; Phirke A. N.; Nadar S. S. Magnetic nanoflowers: a hybrid platform for enzyme immobilization. Crit. Rev. Biotechnol. 2023, 1–22. 10.1080/07388551.2023.2230518. 37455411

[ref40] Ai J.; Zhang W.; Liao G.; Xia H.; Wang D. Immobilization of horseradish peroxidase enzymes on hydrous-titanium and application for phenol removal. RSC Adv. 2016, 6 (44), 38117–38123. 10.1039/C6RA02397E.

[ref41] Barth A. Infrared Spectroscopy of Proteins. Biochimica et Biophysica Acta (BBA) - Bioenergetics 2007, 1767 (9), 1073–1101. 10.1016/j.bbabio.2007.06.004. 17692815

[ref42] Goormaghtigh E.; Ruysschaert J.-M.; Raussens V. Evaluation of the information content in infrared spectra for protein secondary structure determination. Biophys. J. 2006, 90 (8), 2946–2957. 10.1529/biophysj.105.072017. 16428280 PMC1414549

[ref43] Tavares T. S.; da Rocha E. P.; Esteves Nogueira F. G.; Torres J. A.; Silva M. C.; Kuca K.; Ramalho T. C. Δ-FeOOH as support for immobilization peroxidase: optimization via a chemometric approach. Molecules 2020, 25 (2), 259 10.3390/molecules25020259. 31936386 PMC7024332

[ref44] Ulu A.; Noma S. A. A.; Koytepe S.; Ates B. Magnetic Fe_3_O_4_@MCM-41 core–shell nanoparticles functionalized with thiol silane for efficient L-asparaginase immobilization. Artif. Cells Nanomed. Biotechnol. 2018, 46, 1035–1045. 10.1080/21691401.2018.1478422. 29873527

[ref45] Bakar B.; Birhanlı E.; Ulu A.; Boran F.; Yeşilada Ö.; Ateş B. Immobilization of *trametes trogii* laccase on polyvinylpyrrolidone-coated magnetic nanoparticles for biocatalytic degradation of textile dyes. Biocatal. Biotransform. 2024, 42, 194–211. 10.1080/10242422.2023.2173006.

[ref46] Tarhan T.; Dik G.; Ulu A.; Tural B.; Tural S.; Ateş B. Newly synthesized multifunctional biopolymer coated magnetic core/shell Fe_3_O_4_@Au nanoparticles for evaluation of l-asparaginase immobilization. Top. Catal. 2023, 66 (9), 577–591. 10.1007/s11244-022-01742-y.

[ref47] Bordbar A. K.; Rastegari A. A.; Amiri R.; Ranjbakhsh E.; Abbasi M.; Khosropour A. R. Characterization of modified magnetite nanoparticles for albumin immobilization. Biotechnol. Res. Int. 2014, 2014, 705068 10.1155/2014/705068. 24963410 PMC4054909

[ref48] Noma S. A. A.; Ulu A.; Koytepe S.; Ateş B. Preparation and characterization of amino and carboxyl functionalized core-shell Fe_3_O_4_/SiO_2_ for L-Asparaginase immobilization: a comparison study. Biocatal. Biotransfor. 2020, 38 (5), 392–404. 10.1080/10242422.2020.1767605.

[ref49] Yasar U.; Ulusal F.; Huri P. Y.; Guzel B.; Dikmen N. Development of biomaterial-based oxygen transportation vehicles for circulation within blood. J. King Saud. Univ. Sci. 2023, 35 (5), 102689 10.1016/j.jksus.2023.102689.

[ref50] Gül O. T.; Ocsoy I. Preparation of magnetic horseradish peroxidase-laccase nanoflower for rapid and efficient dye degradation with dual mechanism and cyclic use. Mater. Lett. 2021, 303, 130501 10.1016/j.matlet.2021.130501.

[ref51] Fu M.; Xing J.; Ge Z. Preparation of laccase-loaded magnetic nanoflowers and their recycling for efficient degradation of bisphenol A. Sci. Total Environ. 2019, 651, 2857–2865. 10.1016/j.scitotenv.2018.10.145. 30463138

[ref52] Carone A.; Emilsson S.; Mariani P.; Désert A.; Parola S. Gold nanoparticle shape dependence of colloidal stability domains. Nanoscale Adv. 2023, 5 (7), 2017–2026. 10.1039/D2NA00809B. 36998666 PMC10044300

[ref53] Rajamanikandan R.; Shanmugaraj K.; Ilanchelian M. Concentration Dependent catalytic activity of glutathione coated silver nanoparticles for the reduction of 4-nitrophenol and organic dyes. J. Clust.Sci. 2017, 28 (3), 1009–1023. 10.1007/s10876-016-1095-7.

[ref54] Du Y.; Gao J.; Kong W.; Zhou L.; Ma L.; He Y.; Huang Z.; Jiang Y. Enzymatic synthesis of glycerol carbonate using a lipase immobilized on magnetic organosilica nanoflowers as a catalyst. ACS Omega 2018, 3 (6), 6642–6650. 10.1021/acsomega.8b00746. 30023956 PMC6044822

[ref55] Mohammad M.; Ahmadpoor F.; Shojaosadati S. A. Mussel-inspired magnetic nanoflowers as an effective nanozyme and antimicrobial agent for biosensing and catalytic reduction of organic dyes. ACS Omega 2020, 5 (30), 18766–18777. 10.1021/acsomega.0c01864. 32775878 PMC7408242

[ref56] Mohamed S. A.; Al-Harbi M. H.; Almulaiky Y. Q.; Ibrahim I. H.; El-Shishtawy R. M. Immobilization of horseradish peroxidase on Fe_3_O_4_ magnetic nanoparticles. Electron. J. Biotechnol. 2017, 27, 84–90. 10.1016/j.ejbt.2017.03.010.

[ref57] Karim Z.; Khan M. J.; Maskat M. Y.; Adnan R. Immobilization of horseradish peroxidase on β-cyclodextrin-capped silver nanoparticles: its future aspects in biosensor application. Prep. Biochem. Biotechnol. 2016, 46 (4), 321–327. 10.1080/10826068.2015.1031389. 25830286

[ref58] Jun L. Y.; Mubarak N. M.; Yon L. S.; Bing C. H.; Khalid M.; Jagadish P.; Abdullah E. C. Immobilization of peroxidase on functionalized MWCNTs-buckypaper/polyvinyl alcohol nanocomposite membrane. Sci. Rep. 2019, 9, 2215 10.1038/s41598-019-39621-4. 30778111 PMC6379398

[ref59] Li X.; Wu Z.; Tao X.; Li R.; Tian D.; Liu X. Gentle one-step co-precipitation to synthesize bimetallic cocu-mof immobilized laccase for boosting enzyme stability and congo red removal. J. Hazard. Mater. 2022, 438, 129525 10.1016/j.jhazmat.2022.129525. 35816800

[ref60] Kalsoom U.; Khalid N.; Ibrahim A.; Ashraf S. S.; Bhatti H. N.; Ahsan Z.; Zdarta J.; Bilal M. Biocatalytic degradation of reactive blue 221 and direct blue 297 dyes by horseradish peroxidase immobilized on iron oxide nanoparticles with improved kinetic and thermodynamic characteristics. Chemosphere 2023, 312, 137095 10.1016/j.chemosphere.2022.137095. 36334735

[ref61] Weber A. C.; da Silva B. E.; Cordeiro S. G.; Henn G. S.; Costa B.; dos Santos J. S. H.; Corbellini V. A.; Ethur E. M.; Hoehne L. Immobilization of commercial horseradish peroxidase in calcium alginate-starch hybrid support and its application in the biodegradation of phenol red dye. Int. J. Biol. Macromol. 2023, 246, 125723 10.1016/j.ijbiomac.2023.125723. 37419265

[ref62] Amaro-Reyes A.; Díaz-Hernández A.; Gracida J.; García-Almendárez B. E.; Escamilla-García M.; Arredondo-Ochoa T.; Regalado C. Enhanced performance of immobilized xylanase/filter paper-ase on a magnetic chitosan support. Catalysts 2019, 9 (11), 966 10.3390/catal9110966.

[ref63] Asgher M.; Wahab A.; Bilal M.; Iqbal H. M. N. N. Delignification of lignocellulose biomasses by alginate–chitosan immobilized laccase produced from trametes versicolor IBL-04. Waste Biomass Valori. 2018, 9 (11), 2071–2079. 10.1007/s12649-017-9991-0.

[ref64] Bilal M.; Asgher M. Sandal reactive dyes decolorization and cytotoxicity reduction using manganese peroxidase immobilized onto polyvinyl alcohol-alginate beads. Chem. Cent. J. 2015, 9 (1), 47 10.1186/s13065-015-0125-0. 26379768 PMC4570624

[ref65] Sahu S.; Shera S. S.; Banik R. M. Enhanced reusability of horseradish peroxidase immobilized onto graphene oxide/magnetic chitosan beads for cost effective cholesterol oxidase assay. Open Biotechnol. J. 2019, 13 (1), 93–104. 10.2174/1874070701913010093.

[ref66] Jiang Y.; Tang W.; Gao J.; Zhou L.; He Y. Immobilization of horseradish peroxidase in phospholipid-templated titania and its applications in phenolic compounds and dye removal. Enzyme Microb. Technol. 2014, 55, 1–6. 10.1016/j.enzmictec.2013.11.005. 24411438

[ref67] Ameri A.; Faramarzi M. A.; Tarighi S.; Shakibaie M.; Ameri A.; Ramezani-Sarbandi A.; Forootanfar H. Removal of dyes by trametes versicolor laccase immobilized on NaY-Zeolite. Chem. Eng. Res. Des. 2023, 197, 240–253. 10.1016/j.cherd.2023.07.014.

[ref68] Aldhahri M.; Almulaiky Y. Q.; El-Shishtawy R. M.; Al-Shawafi W. M.; Salah N.; Alshahrie A.; Alzahrani H. A. H. Ultra-thin 2D CuO nanosheet for HRP immobilization supported by encapsulation in a polymer matrix: characterization and dye degradation. Catal. Lett. 2021, 151 (1), 232–246. 10.1007/s10562-020-03289-7.

[ref69] Xiao F.; Xiao P.; Jiang W.; Wang D. Immobilization of horseradish peroxidase on Fe_3_O_4_ nanoparticles for enzymatic removal of endocrine disrupting chemicals. Environ. Sci. Pollut. Res. 2020, 27 (19), 24357–24368. 10.1007/s11356-020-08824-y. 32306263

[ref70] Zeyadi M.; Almulaiky Y. Q. Amino functionalized metal–organic framework as eco-friendly support for enhancing stability and reusability of horseradish peroxidase for phenol removal. Biomass Convers. Biorefin 2023, 10.1007/s13399-023-04597-9.

[ref71] Abdulaal W. H.; Almulaiky Y. Q.; El-Shishtawy R. M. Encapsulation of HRP Enzyme onto a magnetic Fe_3_O_4_ Np–pmma film via casting with sustainable biocatalytic activity. Catalysts 2020, 10 (2), 181 10.3390/catal10020181.

[ref72] Dadi S.; Temur N.; Gul O. T.; Yilmaz V.; Ocsoy I. In situ synthesis of horseradish peroxidase nanoflower@carbon nanotube hybrid nanobiocatalysts with greatly enhanced catalytic activity. Langmuir 2023, 39 (13), 4819–4828. 10.1021/acs.langmuir.3c00260. 36944167 PMC10077815

[ref73] Li J.; Chen X.; Xu D.; Pan K. Immobilization of horseradish peroxidase on electrospun magnetic nanofibers for phenol removal. Ecotoxicol. Environ. Saf. 2019, 170, 716–721. 10.1016/j.ecoenv.2018.12.043. 30580166

[ref74] Dlamini M. L.; Lesaoana M.; Kotze I.; Richards H. L. Zeolitic imidazolate frameworks as effective crystalline supports for aspergillus-based laccase immobilization for the biocatalytic degradation of carbamazepine. Chemosphere 2023, 311, 137142 10.1016/j.chemosphere.2022.137142. 36347352

[ref75] Bai J.; Ma H.; Fan X.; Yang H.; Liu C.; Xu Z.; Liu Y. Efficient immobilization of glucose oxidase on mesoporous MIL-125 and their catalytic activities. Results Mater. 2022, 14, 100267 10.1016/j.rinma.2022.100267.

[ref76] Ruxandra Leontieş A.; Rǎducan A.; Cristina Culiţǎ D.; Alexandrescu E.; Moroşan A.; Eduard Mihaiescu D.; Aricov L. Laccase immobilized on chitosan-polyacrylic acid microspheres as highly efficient biocatalyst for naphthol green b and indigo carmine degradation. Chem. Eng. J. 2022, 439, 135654 10.1016/j.cej.2022.135654.

[ref77] Ulusal F.; Özdemir N. Synthesis and characterization of novel mesoporous Fe_3_O_4_ nanotubes for drug delivery. OKU J. Inst. Sci. Technol. 2023, 6 (2), 1353–1368.

[ref78] Bradford M. M. A rapid and sensitive method for the quantitation of microgram quantities of protein utilizing the principle of protein-dye binding. Anal. Biochem. 1976, 72 (1–2), 248–254. 10.1016/0003-2697(76)90527-3. 942051

[ref79] Cheon H. J.; Adhikari M. D.; Chung M.; Tran T. D.; Kim J.; Kim M. Il. Magnetic nanoparticles-embedded enzyme-inorganic hybrid nanoflowers with enhanced peroxidase-like activity and substrate channeling for glucose biosensing. Adv. Healthcare Mater. 2019, 8 (9), 1801507 10.1002/adhm.201801507. 30848070

[ref80] Bilal M.; Iqbal H. M. N.; Hu H.; Wang W.; Zhang X. Enhanced bio-catalytic performance and dye degradation potential of chitosan-encapsulated horseradish peroxidase in a packed bed reactor system. Sci. Total Environ. 2017, 575, 1352–1360. 10.1016/j.scitotenv.2016.09.215. 27720596

