# Supplementary material for: Horseradish Peroxidase Immobilized onto Mesoporous Magnetic Hybrid Nanoflowers for Enzymatic Decolorization of Textile Dyes: A Highly Robust Bioreactor and Boosted Enzyme Stability
Source: ACS Omega. 2024 May 29;9(23):24558–73. doi: 10.1021/acsomega.4c00703 (PMC11170722; doi:10.1021/acsomega.4c00703)
Supplement: Supplementary file 1 — ao4c00703_si_001.pdf [file ao4c00703_si_001.pdf]

Supplementary Information

Horseradish peroxidase-immobilized onto mesoporous magnetic hybrid nanoflowers for enzymatic decolorization of textile dyes: A highly robust bioreactor and boosted enzyme stability

Büşra Bakar[a], Mustafa Akbulut[b], Fatma Ulusal[c], Ahmet Ulu[a*], Nalan Özdemir[b*], Burhan Ateş[a*]

[a]*Biochemistry and Biomaterials Research Laboratory, Department of Chemistry, Faculty of Arts and Science, İnönü University, 44280, Malatya, Türkiye*

[b]*Department of Chemistry, Faculty of Science, Erciyes University, Kayseri, Türkiye*

[c]*Department of Chemistry and Chemical Process Technologies, Vocational School of Technical Sciences, Tarsus University, 33400, Mersin, Türkiye*

Table of contents

Figure S1. BET isotherm of Fe_3O_4–NH_2 NP S3

Figure S2. HR-TEM image of APTES coated mesoporous magnetic Fe_3O_4–NH_2 NPs S4

Figure S3. EDS elemental mapping images of the HRP@Fe_3O_4–NH_2/hNFs S5

Figure S4. Elemental composition of the HRP@Fe_3O_4–NH_2/hNFs after use in the biodegradation of MO (a), PR (b), and MB (c) S6

Figure S5. The effect of organic solvents on the activity of the HRP@Fe_3O_4–NH_2/hNFs S7

Figure S6. The chemical structures of MO, PR, and MB dyes S8

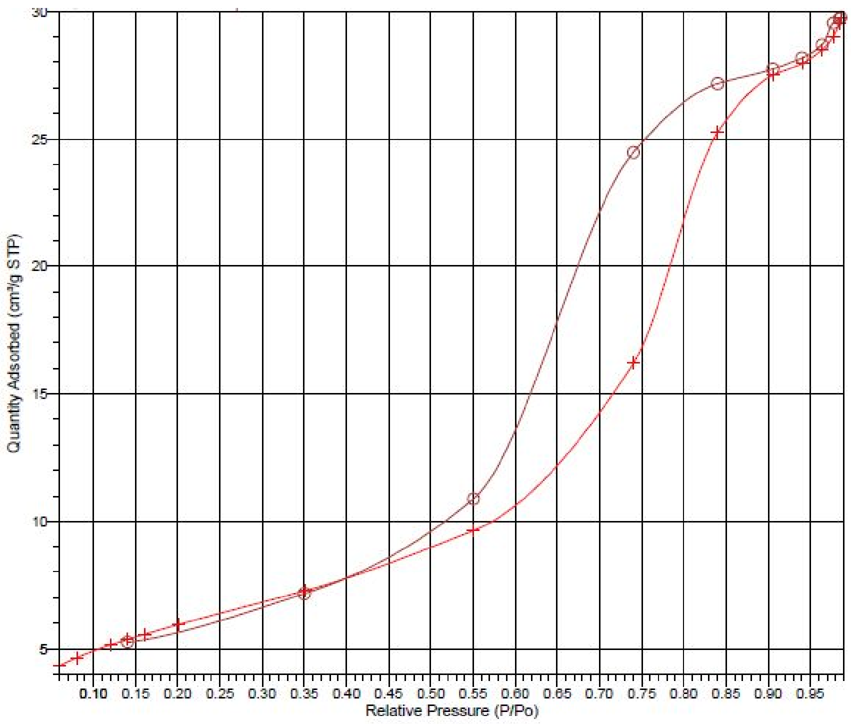

Figure S1. BET isotherm of Fe_3O_4–NH_2 NPs.

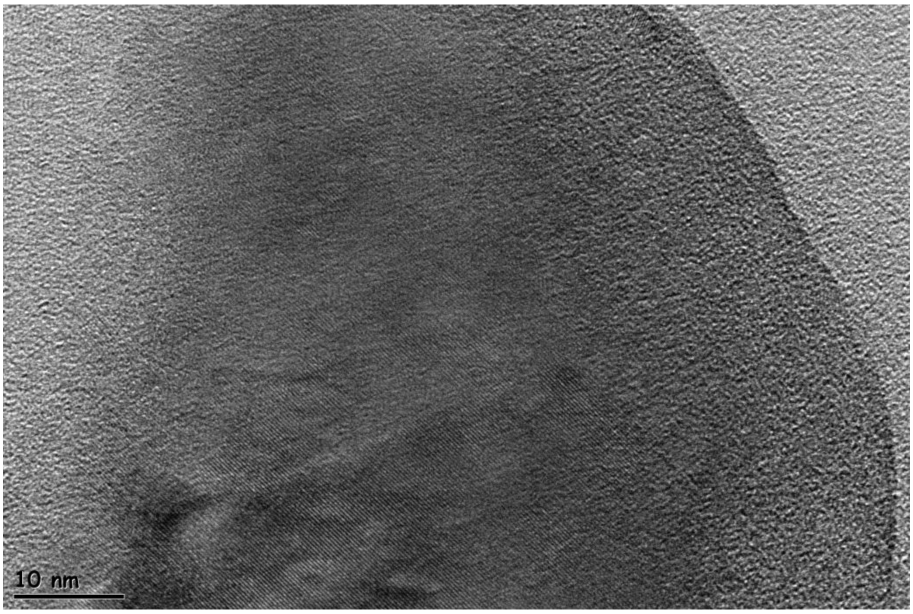

Figure S2. HR-TEM image of APTES coated mesoporous magnetic Fe_3O_4–NH_2 NPs.

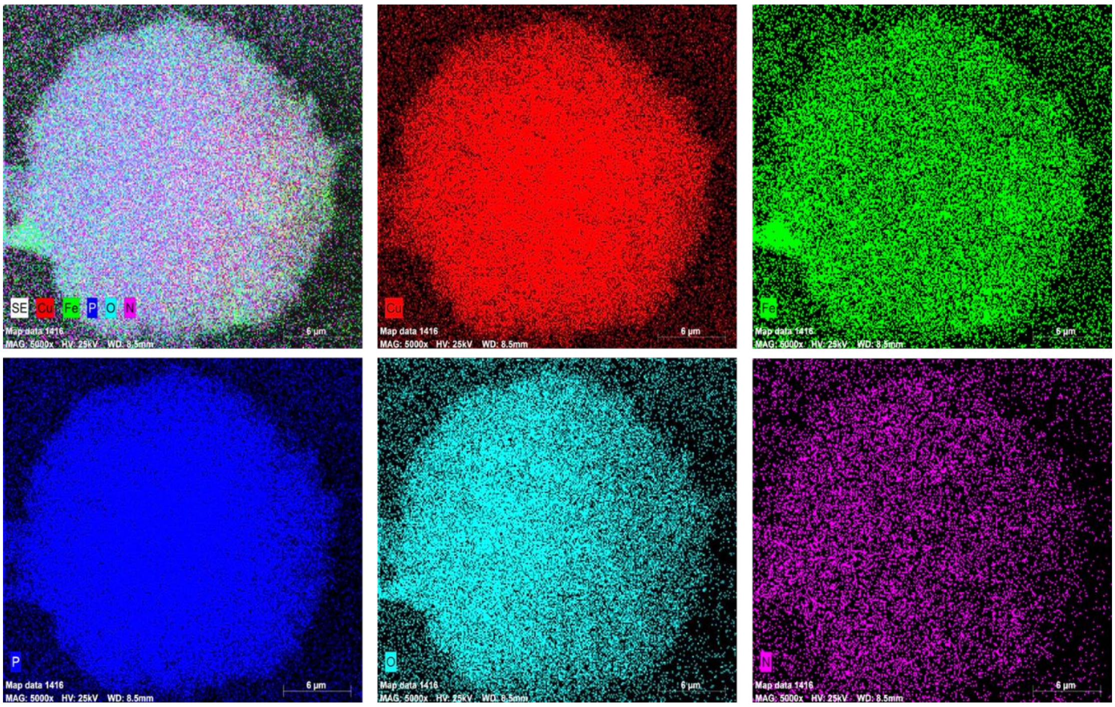

Figure S3. EDS elemental mapping images of the HRP@Fe_3O_4–NH_2/hNFs.

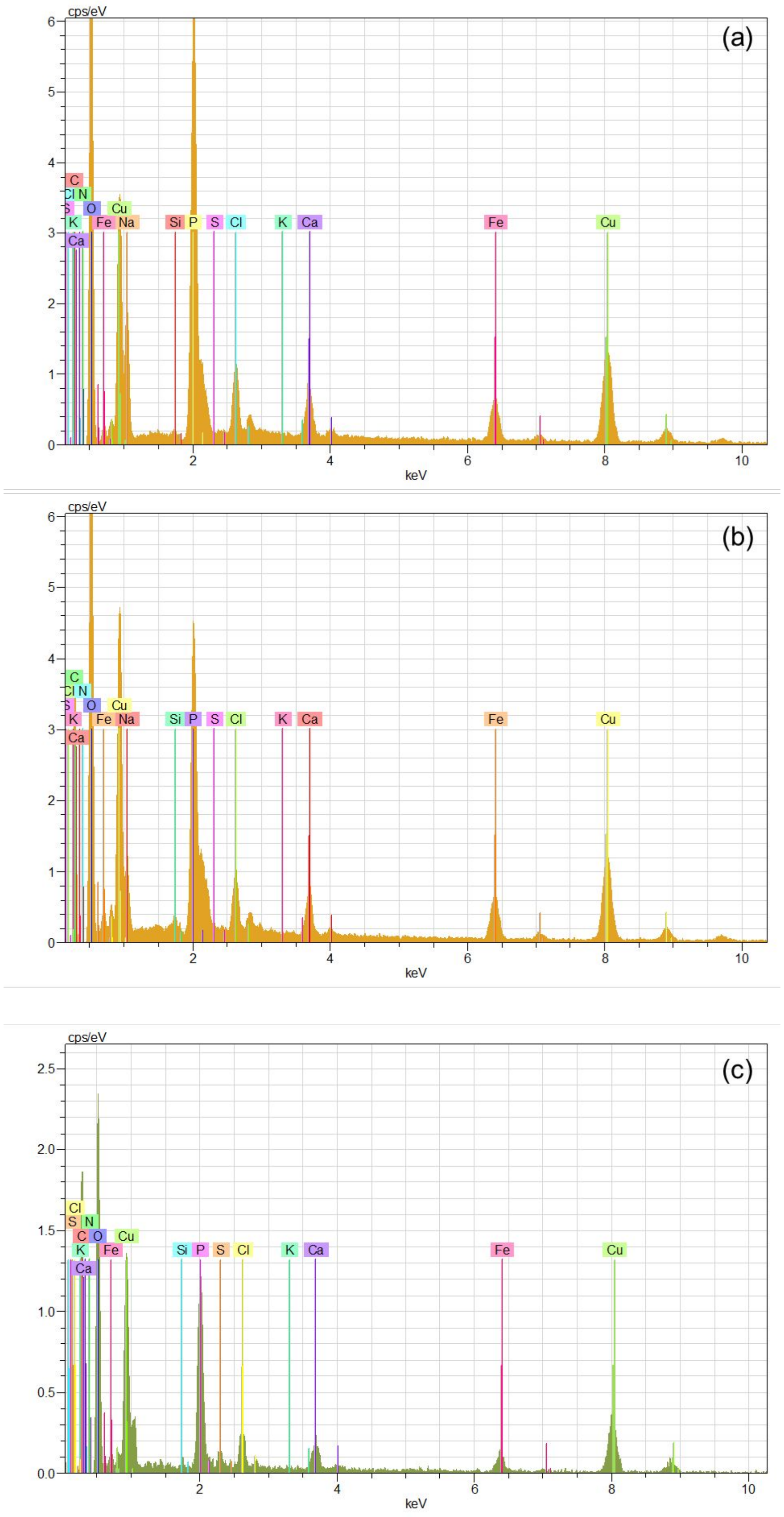

Figure S4. Elemental composition of the HRP@Fe_3O_4–NH_2/hNFs after use in the biodegradation of MO (a), PR (b), and MB (c).

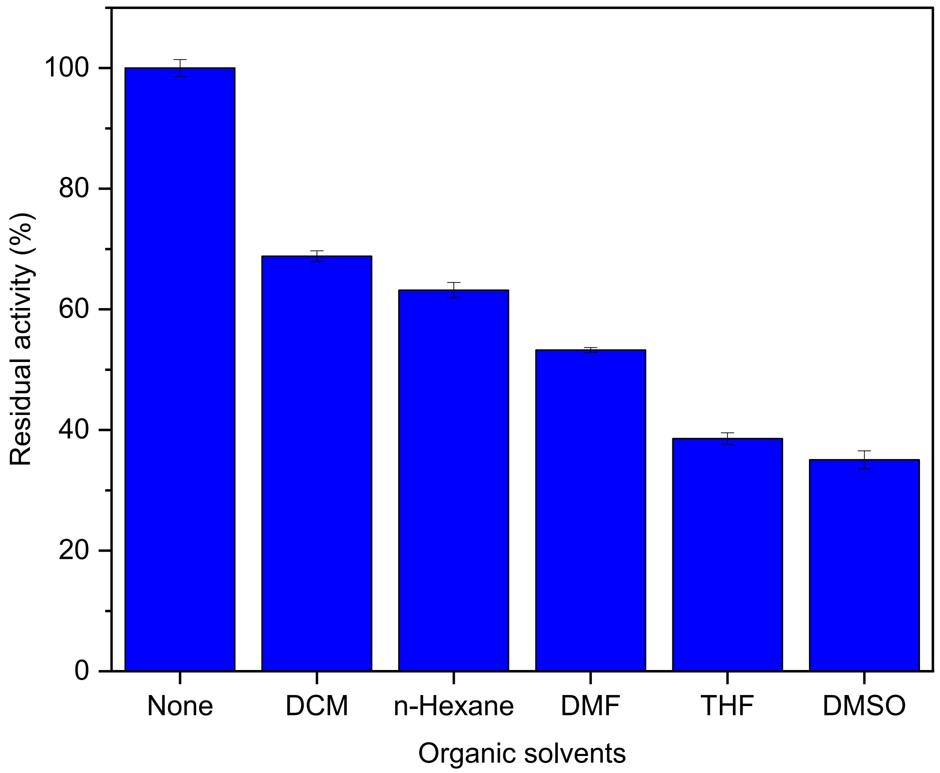

Figure S5. The effect of organic solvents on the activity of the HRP@Fe_3O_4–NH_2/hNFs.

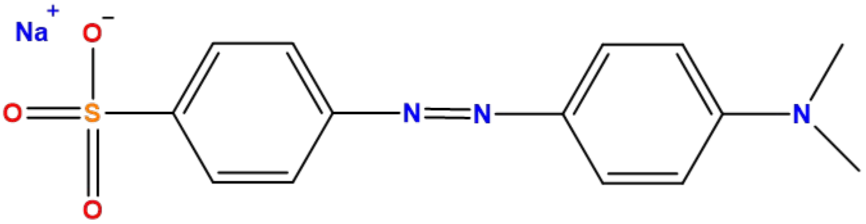

Methyl Orange

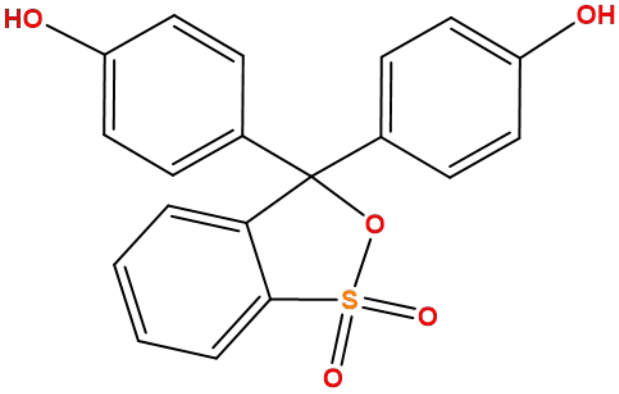

Phenol Red

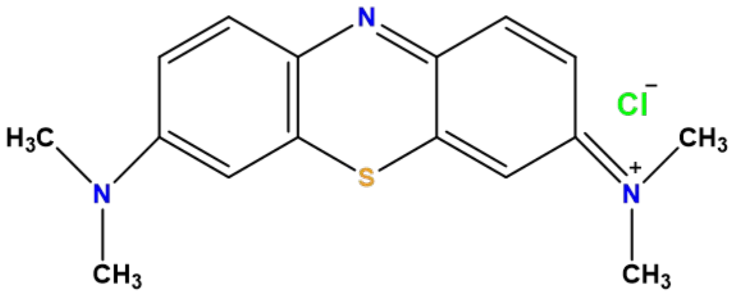

Methylene Blue

Figure S6. The chemical structures of MO, PR, and MB dyes.
